# The two-domain elevator-type mechanism of zinc-transporting ZIP proteins

**DOI:** 10.1126/sciadv.abn4331

**Published:** 2022-07-13

**Authors:** Anders Wiuf, Jonas Hyld Steffen, Eva Ramos Becares, Christina Grønberg, Dhani Ram Mahato, Søren G. F. Rasmussen, Magnus Andersson, Tristan Croll, Kamil Gotfryd, Pontus Gourdon

**Affiliations:** ^1^ Department of Biomedical Sciences, University of Copenhagen, Mærsk Tower 7-9, Nørre Allé 14, DK-2200 Copenhagen, Denmark.; ^2^ Department of Chemistry, Umeå University, Linnaeus Väg 10, SE-901 87 Umeå, Sweden.; ^3^ Department of Neuroscience, University of Copenhagen, Maersk Tower 7-5, Nørre Allé 14, DK-2200 Copenhagen, Denmark.; ^4^ Cambridge Institute for Medical Research, Department of Haematology, University of Cambridge, Keith Peters Building, Hills Rd., Cambridge CB2 0XY, UK.; ^5^ Department of Experimental Medical Science, Lund University, Sölvegatan 19, SE-221 84 Lund, Sweden.

## Abstract

Zinc is essential for all organisms and yet detrimental at elevated levels. Hence, homeostasis of this metal is tightly regulated. The Zrt/Irt-like proteins (ZIPs) represent the only zinc importers in metazoans. Mutations in human ZIPs cause serious disorders, but the mechanism by which ZIPs transfer zinc remains elusive. Hitherto, structural information is only available for a model member, BbZIP, and as a single, ion-bound conformation, precluding mechanistic insights. Here, we elucidate an inward-open metal-free BbZIP structure, differing substantially in the relative positions of the two separate domains of ZIPs. With accompanying coevolutional analyses, mutagenesis, and uptake assays, the data point to an elevator-type transport mechanism, likely shared within the ZIP family, unifying earlier functional data. Moreover, the structure reveals a previously unknown ninth transmembrane segment that is important for activity in vivo. Our findings outline the mechanistic principles governing ZIP-protein transport and enhance the molecular understanding of ZIP-related disorders.

## Teaser

Structure of a zinc importer in a novel state sheds new light on the molecular determinants of zinc homeostasis.

## INTRODUCTION

Zinc (Zn^2+^) is essential for all domains of life. It is the second most abundant trace metal in humans (*1*), required for the function of more than 300 enzymes and 1000 transcription factors (*1*, *2*). Consequently, zinc dyshomeostasis is linked to a broad palette of diseases, including diabetes, immune disorders, and progression of several forms of cancer (*1*, *3*). The Zrt/Irt-like protein (ZIP) family of solute carrier 39A (SLC39A) membrane transporters is critical in maintaining appropriate cellular levels of zinc. ZIPs represent the only identified zinc importers in mammals, providing metal transfer from the extracellular space or from internal storage vesicles to the cytoplasm (*3*). As expected, malfunction of the human ZIPs, hZIPs, is associated with development of different disorders, e.g., hZIP4 and hZIP13 are directly linked to the severe acrodermatitis enteropathica and the spondylocheiro dysplastic form of Ehlers-Danlos syndrome, respectively (*4*, *5*).

However, the structure and function of ZIPs remain elusive, preventing applied efforts to combat Zn^2+^ imbalance or directly related disorders as well. Both Michaelis-Menten kinetics, typical for secondary active transporters, and channel-like mechanisms have been proposed for ZIPs. Zn^2+^/HCO_3_^−^ (*6*–*10*) and Zn^2+^/H^+^ symport (*11*) or even passive transport (*12*) have been detected for human members. Moreover, the ZIP from *Bordetella bronchiseptica*, BbZIP, has been suggested to display properties of nonsaturable electrogenic diffusion, operating as a channel (*13*). Similarly, although ZIPs are known to assemble as homodimers, the monomer-monomer arrangement is debated (*14*–*16*). Architectural information for ZIPs is limited to data on BbZIP and an isolated soluble domain of hZIP4 (*16*–*18*). The identified ZIP fold is unique and consists primarily of a transmembrane domain of eight pseudo-symmetrically arranged transmembrane segments (TMs) 1 to 8. Together, they form two separate helix bundles encompassing TM1, TM4, TM5, and TM6 and TM2, TM3, TM7, and TM8, respectively. Furthermore, a central ion-binding region was identified between the two bundles, with the metal coordinated by residues of TM4 and TM5 (*16*). However, the available BbZIP structures represent a single inward-open, outward-closed, and metal-bound state (with interacting Zn^2+^ and/or Cd^2+^), in agreement with properties of transporters and, possibly, gated channels. In this configuration, a substantial cavity between the two bundles provides access to the metal-binding region from the intracellular side. In contrast, the putative entry pathway is blocked by highly conserved hydrophobic residues. Consequently, the operational principles of ZIPs represent an outstanding question; even the overall classification as transporters or channels remains elusive. The high sequence homology among members suggests a shared overall shape and maintained mechanistic features throughout the entire ZIP family. Nevertheless, a multitude of transport modes is still permitted.

Here, aiming to elucidate how Zn^2+^ traverses cellular membranes across ZIP proteins, we determined the structure of BbZIP in a previously uncharacterized conformation together with in silico and in vivo analyses. The findings point to a shared transport mechanism for the entire family of ZIP proteins.

## RESULTS AND DISCUSSION

### Structure of BbZIP in metal-free condition reveals a previously unidentified inward-open state

**Fig. 1.**
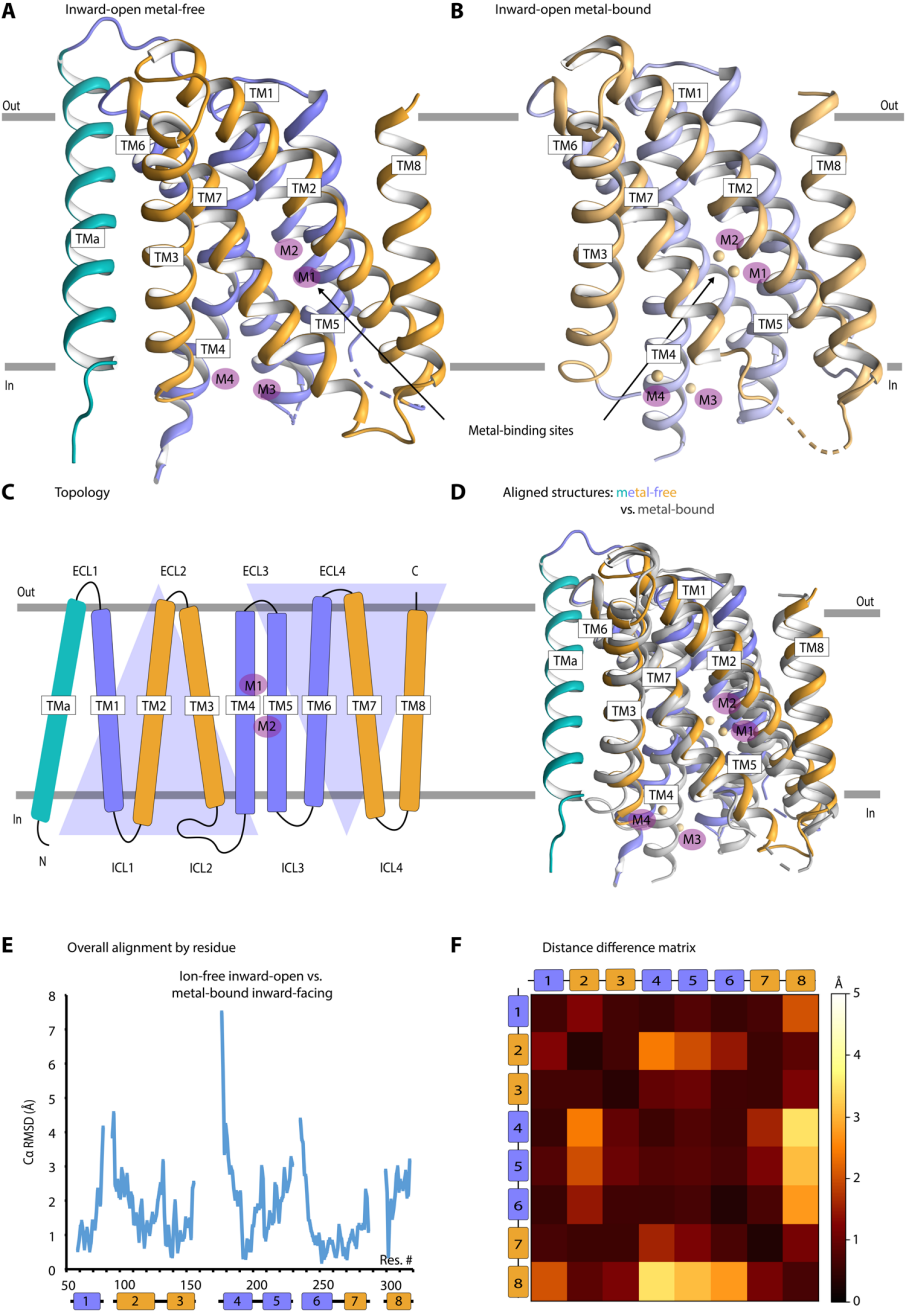
Structure of BbZIP in an inward-open metal-free state reveals bundle movements. (**A**) Side view of the inward-open metal-free structure without the presence of ions in metal-binding sites (M1, M2, M3, and M4 shown as purple spheres throughout). The structure reveals an additional N-terminal transmembrane segment (TMa in cyan). TM1, TM4, TM5, and TM6 constitute the transport domain (purple), while TM2, TM3, TM7, and TM8 form the scaffold domain (orange). **(B**) Side view of the inward-open metal-bound structure. Bound Cd^2+^ ions are shown as purple spheres. (**C**) Topology of ZIPs including the previously unidentified helix (TMa) present in certain ZIP members. Purple triangles indicate symmetry. (**D**) Overall alignment of C_α_ atoms in the metal-bound and metal-free states (chain B), revealing overall similarity and yet prominent displacements. (**E**) Comparison of pairwise root mean square deviation (RMSD) values for the overall alignment of C_α_ reveals areas with substantial differences between the two conformations, particularly the cytoplasmic parts of TM1, TM4, TM5, and TM6. (**F**) Distance difference matrix demonstrating unbiased helix movements, calculated using a previously reported script (https://github.com/GaudetLab/archived-DDMP/) (*50*). The matrix was generated through calculation of pairwise C_α_-C_α_ distances for each structure (TMa was not included) and, subsequently, by applying matrix subtraction to compare distances. A helix pair that remains fixed relative to each other will have lower RMSD value and is represented with dark color in the heatmap. Conversely, helix pairs having relatively high mobility are characterized by higher RMSD values and lighter colors. The scale bar indicates the RMSD differences (in Å) by TMs.

To further dissect the function of ZIP transporters, we sought to structurally determine the enigmatic ion-free form. Because even prokaryotic members of the SLC39A family are exceptionally difficult to isolate (*13*), BbZIP serves as the only identified suitable homolog for structural and functional studies of a full-length ZIP. Previous studies performed at pH 7.3 have indicated that BbZIP is intolerant to the absence of metals (*13*, *16*). We hypothesized that pH reduction would metal strip BbZIP and found that the protein is stable over weeks at pH 4.5 in the absence of Zn^2+^ or its congener Cd^2+^ in the buffer. With such treated sample, we crystallized BbZIP in the lipidic cubic phase (LCP) using metal-free conditions at pH 4.5. The resulting crystals diffracted anisotropically to 2.6 to 3.6 Å, and the structure was readily solved by molecular replacement (Fig. 1A, table S1, and fig. S1). Notably, even initial analysis disclosed that the new structure is metal-free, as the metal-binding region is void of density or anomalous signal from bound cation. In addition, no difference peaks corresponding to an unmodeled ion appeared in the final model (fig. S1). In contrast, separate reproduction of the previously reported Cd^2+^-bound form (henceforth referred to as metal bound), here determined at 2.5 Å resolution, pH 7.4, reveals metal presence using the same criteria (Fig. 1B, table S1, and fig. S2). Overall, the metal-bound and metal-free structures are reminiscent (Fig. 1D), each displaying an inward-open configuration of TM1 to TM8. However, the latter uncovers an additional, approximately 30-residue-long and tilted transmembrane segment (here termed TMa) in the N terminus (Fig. 1, A and C). This region was missing in previously reported structures. Our crystallographic data and accompanying sequence analyses now clearly demonstrate that the ZIP fold can accommodate an additional helix, with potentially important consequences for cellular Zn^2+^ import (see more below and fig. S3).

### Crystal packing in metal-free BbZIP crystals, coupled with coevolution analysis, uncover the BbZIP dimer interface

**Fig. 2.**
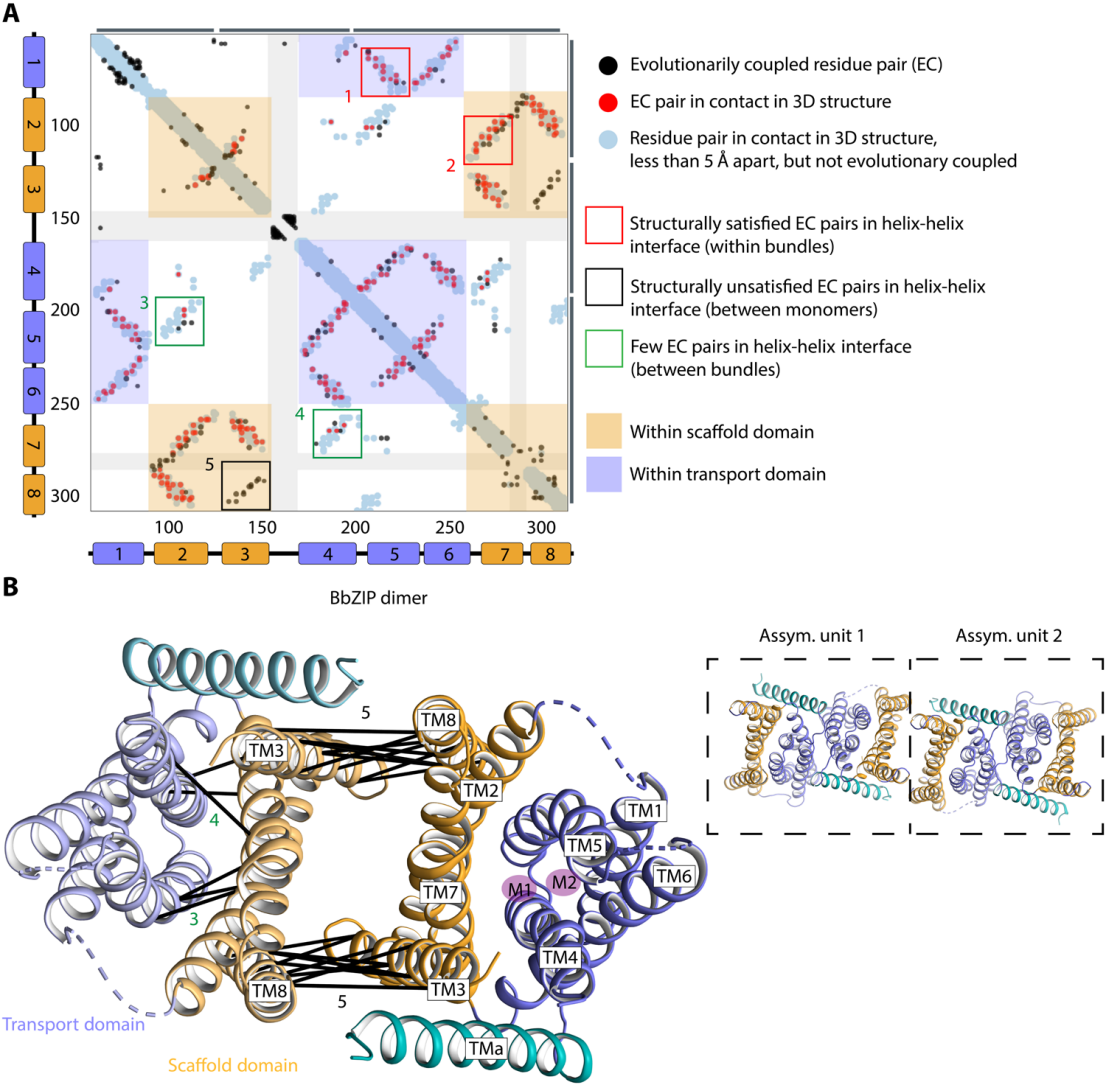
Evolutionary covariation analysis of ZIPs. (**A**) Analysis of evolutionary coupled amino acids was performed using the EVCS (https://evcouplings2.hms.harvard.edu/) (*19*). TMa is omitted because of lack of sequence information in the alignment. The resulting contact map shows predicted evolutionary pairs (black dots) and true structural contacts (blue dots). Overlaps of EVCS-predicted constraints and monomer-available structural contacts are shown as red dots. Interactions between α-helix interfaces result in diagonal lines. Interfaces can be either parallel (as for TM4 and TM6, illustrated as a “\”-shape) or antiparallel (TM1 and TM6, illustrated as a “/”). Examples of helix-helix interactions, typically present within the bundles, are highlighted by the red boxes (1 and 2). Green boxes (3 and 4) contain monomer-available helix interfaces with structural contacts, but few are predicted by EVCS, as they are generally available between the bundles. Strong evolutionary pairs are only found within helix bundles, whereas the intrabundle helix interfaces are almost devoid of evolutionary coupled pairs. Black box, 5, indicates helix interactions not found in the monomer, which are satisfied by the proposed dimer. (**B**) Evolutionary coupled pairs are connected by black lines in the BbZIP structure. The numbers of the connections refer to the example boxes in (A). The crystal packing (insert, right) of the inward-open metal-free structure displays an interface between two asymmetric units that matches the EVCS-predicted antiparallel helix interaction between TM3 and TM8. This arrangement agrees with a homodimer supported by the scaffold domain. A few evolutionary coupled residues are also found in between the two bundles. See also fig. S5. 3D, three-dimensional.

It is well established that ZIPs form dimers, as is also valid for detergent-purified BbZIP (*16*). The significance of this assembly is underscored by the fact that severe disease-causing mutations of hZIP4 have been suggested to influence dimerization (*18*). Nevertheless, the molecular details of the dimer assembly remain elusive. This is partly because the available metal-bound structures exhibit an antiparallel (i.e., inside-in/inside-out) crystal packing. Thus, these structures do not provide insight into the arrangement of ZIP dimers in native membranes. By investigating the crystal packing in our metal-free BbZIP structure, we identify two separate dimers displaying a parallel (i.e., inside-in/inside-in) crystal packing. One of these occurs within the asymmetric unit, while the other dimer is present in between two asymmetric units (fig. S4). To further investigate the native dimer architecture, we performed analysis of coevolved residues among ZIPs using the EVcouplings server (EVCS) (*19*). Assuming that structurally and/or functionally important interfaces are more coevolved than other surfaces, such evaluation can identify oligomeric interactions (Fig. 2). The generated contact map is rich in information for the ZIP family, most consistent with the BbZIP structures, with the general trend of intrahelix-bundle interactions being dominating (Fig. 2A and fig. S5; red boxes/red lines indicate examples). However, the calculated map illustrates some contacts not explained by the BbZIP monomer. In particular, there is a clear interaction between TM3 and TM8 (Fig. 2, A and B; black box/lines), two helices that, in the monomer, are more than 20 Å apart. This interface is only possible through integration of helices of a second monomer, resulting in a convincing dimer with a dimer-interface in-between two asymmetric units in the crystal packing (Fig. 2B and fig. S4). With this arrangement, all predicted contacts between TM3 and TM8 are fulfilled, strengthening the notion of a TM3/TM8-induced oligomerization of ZIPs (fig. S4).

Furthermore, surface conservation analysis of ZIP proteins is congruent with the identified dimer (fig. S6A), and the ColabFold software (*20*) predicts a highly similar dimer arrangement but with a tighter packing (fig. S7). Moreover, because of the latter arrangement, TM7 and TM8 from opposite protomers come in close proximity. Such an arrangement explains the evolutionary coupled residues between TM7 and TM8 in the coevolution analysis (Fig. 2A). To further confirm the validity of this dimer interface, we conducted computational docking of BbZIP monomers. Using the prediction analysis of microarrays method (*21*), 100 docking poses were identified as six clusters (fig. S6B). The cluster with the highest population of docked monomers (fig. S6C) is congruent with the proposed dimer interface with a backbone root mean square deviation (RMSD) of 3.3 ± 0.8 Å (fig. S6D). Notably, these data also agree with what has been earlier proposed for hZIP2 and hZIP4, respectively (*12*, *22*), and thus suggest validity for the entire ZIP family.

### ZIP helix bundles rearrange as rigid bodies compatible with an alternating-access mechanism

Detailed structural comparisons of the metal-free and metal-bound inward-open structures expose notable differences for one of the two protomers (chain B), with an overall RMSD of 1.75 Å (hereafter referred to as the ion-free structure; Fig. 1E). To further and objectively dissect the observed variance, we generated a C_α_-to-C_α_ distance difference matrix. Here, the calculated C_α_-to-C_α_ distances for each of the states were subtracted and then averaged by TM helix (Fig. 1F) (*23*). Notably, helix bundle–dependent shifts are detected by our analysis. Helices within the two bundles (i.e., TM1, TM4, TM5, and TM6 and TM2, TM3, TM7, and TM8, respectively) are generally not displaced relative to each other. Conversely, the helices display larger movements between the bundles, particularly in between TM4, TM5, and TM6 and TM2 and TM8, respectively. Collectively, these observations indicate that the bundles move as rigid bodies relative to each other in a Zn^2+^-dependent manner. This suggests that we have captured a previously unidentified structural state of the ZIP proteins. Moreover, this is of significance, as the rocking bundle and elevator types of alternating-access mechanisms both achieve cargo transport through rigid body–like movements (*24*, *25*). Hence, our observation may hint at the modus operandi of ZIP proteins. Our BbZIP structure displays many features that are congruent with an alternating-access mechanism. These include the two clearly distinct helix bundles with inverted repeat symmetry, a central metal-binding region, and, last, an inward-open state, with no obvious transport path or uptake region on the opposite side, as also reported earlier (*16*).

### ZIPs exhibit hallmarks of two-domain elevator transporters

Evolutionary coupling may also hint at the mechanism type of membrane protein transporters. Specifically, the interface between the two bundles in well-established elevator-type transporters (UraA, UapA, GltPh, and BicA) are either lacking or have few residues that coevolve (fig. S8). In contrast, the domain interface of “rocker switch” proteins, such as the glucose transporters, have many amino acids that coevolve (*26*). Similarly, the bundle interface of “rocking-bundle” transporters, such as LeuT, also include multiple residues that coevolve. Because the bundle interface of BbZIP proteins has few residues that coevolve (Fig. 2), it is likely that ZIP proteins transport metals using an elevator-type mechanism.

**Fig. 3.**
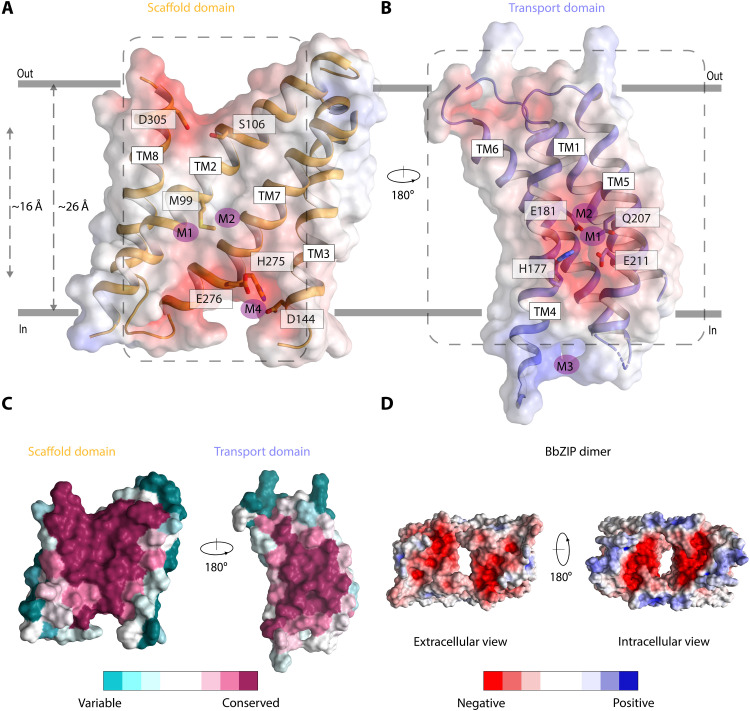
Structural hallmarks of the two-domain elevator-type transport mechanism of ZIPs. (**A**) Transparent electrostatic view of the scaffold domain (orange) as seen from the transport domain (dotted lines). Symmetrically related inverted V repeats created by TM2/8 and TM3/7 in the scaffold domain are visible. Two tilted antiparallel helices (TM2 and TM7) create a hydrophobic barrier (~16 Å) that is narrower than the membrane (~26 Å). Residues of the putative entry (S106 and D305) and release (D144 and H275) pathways are nestled inside each V motif. All residues are shown as sticks. The metal-binding sites (M1 to M4) from the metal-bound structure are shown as spheres. (**B**) Transparent electrostatic surface view of the transport domain (purple) as seen from the scaffold domain (dotted lines). The transport domain includes the central ion-binding region (M1 and M2 in metal-bound structure of BbZIP) for which the electrostatic potential is highly negative in the absence of zinc. Zinc-coordinating residues (H177, E181, Q207, and E211) are shown as sticks. (**C**) Surface conservation representations of the equivalent surfaces as shown in (A) and (B) generated using the ConSurf Server (https://consurf.tau.ac.il/) (*55*). The ion-binding region of the transport domain and the hydrophobic character of the scaffold domain, respectively, are highly conserved. (**D**) Electrostatic surface representations of the proposed dimer as observed from the outside and inside of the cell, respectively, demonstrating shared regions for ion uptake and release, the latter representing a classical elevator feature.

A hallmark of elevator proteins is that they share a dynamic transport domain that exclusively interacts with the cargo and undergoes displacement across the membrane, in addition to a rotational movement during transport. Conversely, a more static scaffold domain serves as the sole interface for obligate oligomerization (*27*). Notably, the scaffold domains of the four elevator-type transporters included in our analysis all align and superimpose well with the aforementioned TM2, TM3, TM7, and TM8 helix bundle of BbZIP, from now on termed the scaffold domain (fig. S9). In contrast, larger differences are found between the transport domains of all the proteins. However, the TM1, TM4, TM5, and TM6 bundle of BbZIP displays key characteristics of elevator transporter domains, as the metal-binding region is formed almost entirely by residues of TM4, TM5, and TM6. Hence, we designate it as the transport domain. In agreement with the notion that ZIPs operate as elevator transporters, the two domains have other properties in common with known elevator-type domains (*25*, *28*). The scaffold domain is generally electrostatically neutral or hydrophobic, as it is also the case for BbZIP (Fig. 3A). In contrast, the transport domain is typically considerably polar, and this is valid also for BbZIP, for which this bundle is negatively charged (Fig. 3B). Furthermore, the scaffold domain of ZIPs shares common topological features exclusive to elevator-type transporters. These characteristics include the symmetrically inverted V-repeats of the TM helices and the presence of long, antiparallel, and tilted central transmembrane segments (consisting of TM2 and TM7 in BbZIP). Together, they reduce the distance (membrane height) that the ions need to travel across the membrane, from approximately 26 to 16 Å through formation of membrane invaginations (Fig. 3A) (*24*). Collectively, these data suggest that ZIPs exploit a two-domain elevator-type mechanism for cellular uptake of Zn^2+^.

### Elevator conformational changes permit Zn^2+^ uptake and release

**Fig. 4.**
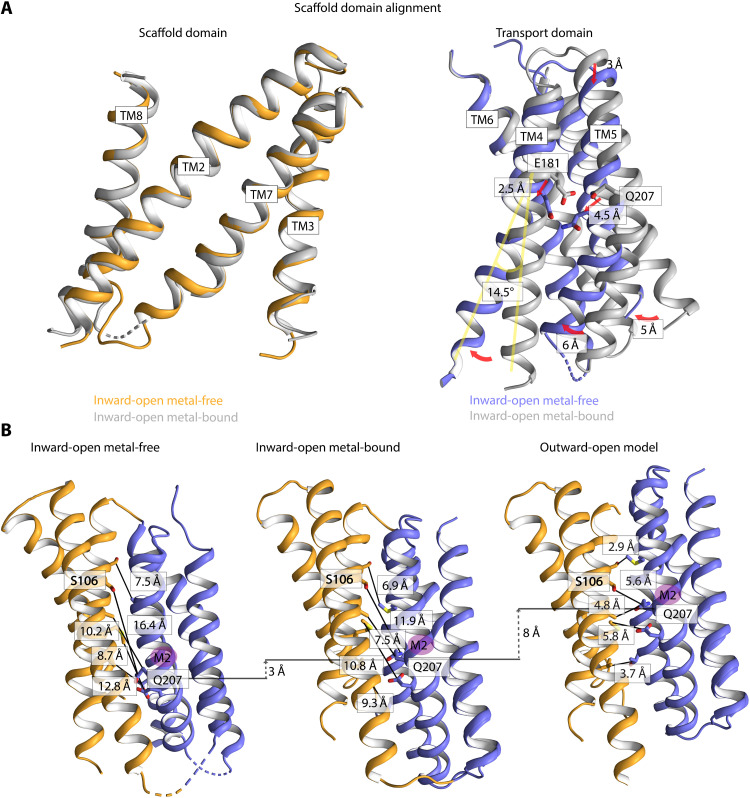
Conformational changes in ZIPs. (**A**) Alignment of metal-bound (gray) and metal-free inward-open states based on the scaffold domain. The transport domain of the inward-open metal-free structure is displaced 3 Å toward the cytoplasm and is tilted 11° relative to the scaffold domain. The metal-binding region site has relocated approximately 4.5 Å. Arrows indicate the largest displacements of helices; the cytoplasmic part of TM4 is rearranged 14.5°. Displacements of the transport domain (blue) and the ion-binding region between the two structures are congruent with an elevator-type mechanism. Zinc-coordinating residues (E181 and Q207) are shown as sticks. The rearrangements have been calculated using the angleBetweenDomains PyMOL script. (**B**) Conformations that permit ion uptake and release. The metal-free and metal-bound conformations represent the structures determined here, while the outward-open state is a generated model. Evolutionary coupled pairs (ECs) in the interdomain area that are unsatisfied in the inward-facing conformations are mapped on the three distinct states. For these ECs to be satisfied, a considerable 8 Å translation, perpendicular to the membrane, of the transport domain is required as fulfilled in the outward-facing model. Noteworthy, these ECs cannot be satisfied by rocking bundle mechanisms. For clarity, TM8 has been omitted in all states. Residue of the putative entry (S106) and zinc-coordinating residue (Q207) are shown as sticks.

To investigate zinc-dependent movement of the transport domain, we aligned the metal-free and metal-bound structures using the static scaffold domains (Fig. 4A). The analysis reveals a considerable displacement of the transport domain, with the ion-free form being shifted around 3 Å toward the intracellular inside, combined with a substantial twist that tilts the domain 11°. Thereby, the Zn^2+^-binding region is directly exposed to the cytoplasm, in agreement with completion of ion release during the course of a transport cycle (Figs. 3, C and D; 4B; and 5).

**Fig. 5.**
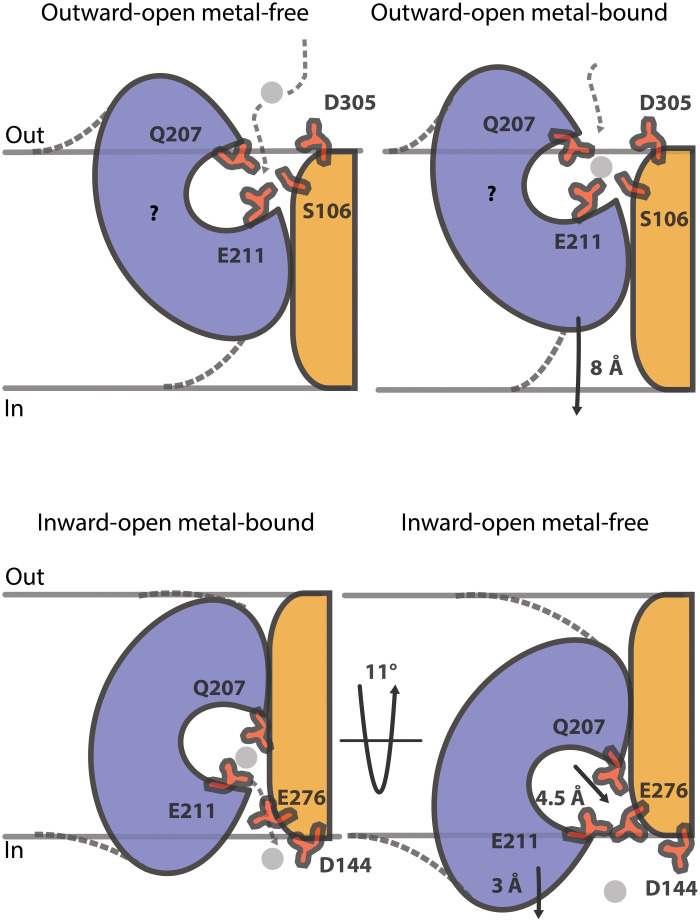
The elevator transport mechanism of ZIPs. The proposed transport mechanism with functionally important residues marked in red. The dimer forms a stable scaffold that allows the transport domains to oscillate between the states. The ion-binding region of the transport domain is alternatively exposed to functional residues of the scaffold domain linked to the cellular outside and inside, respectively. Zinc ions (shown as gray spheres) are recruited to the negatively charged area around S106, Q207, E211, and D305. It is likely that in a hypothetical outward-facing state, the zinc-coordinating residues in the metal-binding site will move closer toward S106 and D305, as proposed in our outward-open model. Metal binding permits conformational changes, most notably a distinct elevator-like translocation across the membrane of the transport domain through the membrane. In the inward-open metal-bound state, release of the ion from the metal-binding region is mediated through zinc-binding residues, including D144, H275, and E276. Last, the structural rearrangement detected here has to occur to fully release bound metal.

Conversely, extracellular access is provided through electronegatively charged residues of TM2, TM3, and TM7. It is even possible that a common bowl covers the two scaffold domains, thereby orchestrating efficient recruitment of metal ions. Notably, evaluation of the few identified coevolved interdomain contacts also hints at a possible outward-facing configuration, as a subset of these are not fulfilled in the determined inward-facing states. Minimization of these distances through stepwise translation of the transport domain across the membrane suggests that an 8 Å rearrangement toward the extracellular side is necessary, relative to the metal-bound inward-facing state (Fig. 4B). The magnitude of this conformational change is comparable with that experimentally observed for other elevator transporters such as the bicarbonate transporter, BicA (*29*), the anion exchanger 1, AE1 (*24*), and the borate efflux transporter, Bor1 (*30*). In addition, the seemingly brief transport displacement is facilitated by the shorter transport barrier created by the scaffold domain (Fig. 3A) (*25*). Notably, such a hypothetical configuration has indeed the potential to surface-expose the ion-binding region via a negatively charged cavity present already in the inward-facing structures (lined by TM2 and TM8).

### A transport mechanism involving conserved residues

We propose that ZIPs form scaffold domain–based dimers and exploit an oscillating elevator-type mechanism. In this way, the metal-binding region would be alternately exposed to symmetrically related negatively charged cavities, which maybe even comprise common entry and exit areas for Zn^2+^ within the dimer (Fig. 5). Considering this, it is tempting to speculate that ZIP oligomerization serves a stabilizing role, rather than partaking in cooperativity and/or (allosteric) regulation. This is also supported by the observation that BbZIP dimerization appears reversible during purification in detergent environment. Regarding uptake, in the Na^+^/H^+^ antiporting elevators NapA and MjNhaP1, the outer surface of the scaffold domain is negatively charged, which has been proposed to enable selection and concentration of the transported cargo (*23*, *24*). In BbZIP, cargo recruitment from the extracellular side may be achieved through the TM2/8 opening in an outward-facing state. This is possibly stimulated by the well-preserved S106 and D305, and the more surface-exposed D113 of the cavity, providing access to E181, E211, and Q207 of the ion-binding region (fig. S10). Next, metal-binding permits a modest rotation and a ~11-Å sliding of the transport domain toward the intracellular side along the inert scaffold domain. Here, Zn^2+^ becomes occluded, bound almost exclusive through side chains of the transport domain. Release is facilitated by the TM2, TM3, and TM7 bowl, lined by the invariant D144 and H275, and E276, as also demonstrated by our metal-bound and metal-free structures. Last, the movement of the entire transport domain reverts to allow a new transport cycle.

**Fig. 6.**
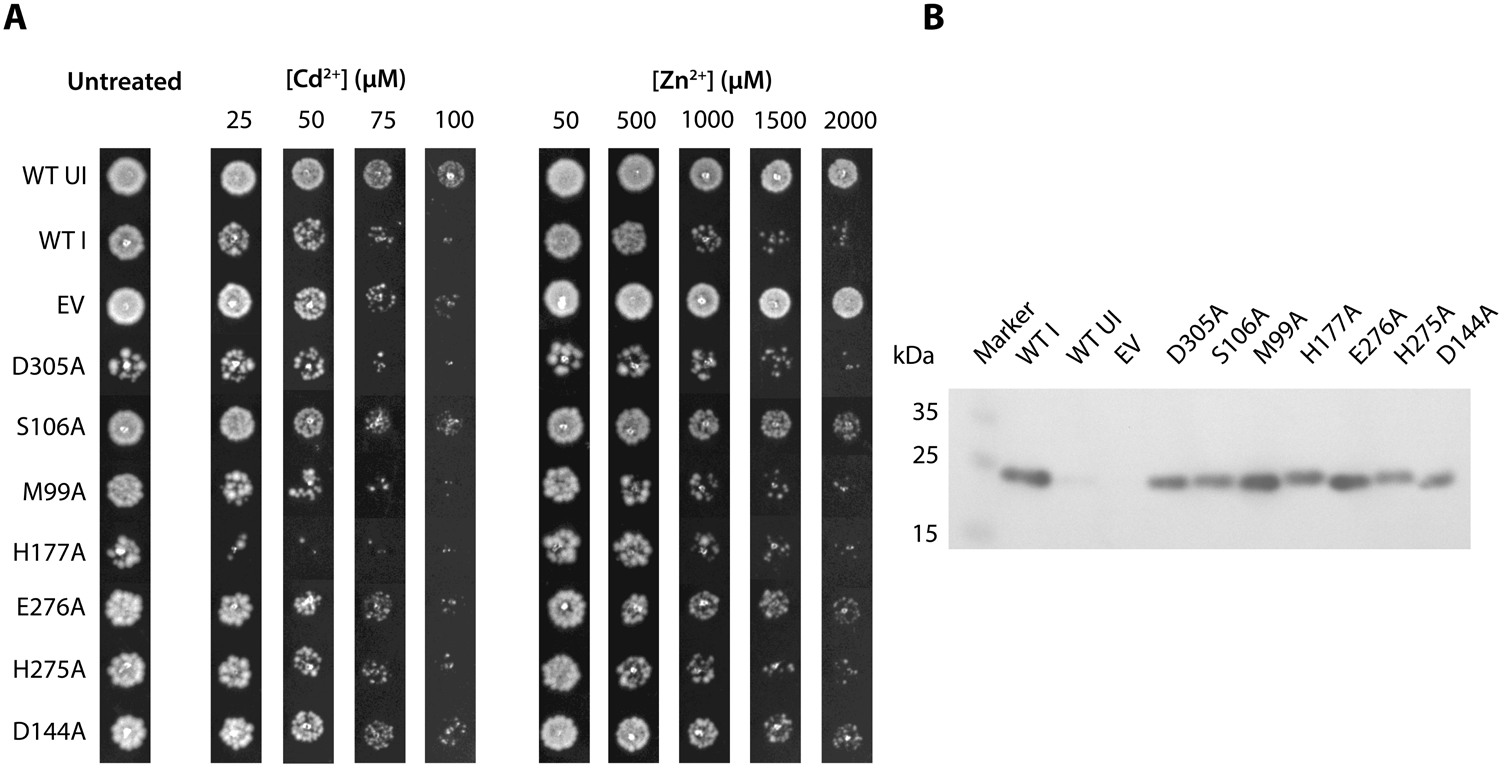
Assessment of conserved residues using an in vivo metal toxicity assay. (**A**) *E. coli* cells [strain C43(DE3)] carrying either wild-type BbZIP (WT) or single-alanine mutations were spotted on LB agar plates containing the indicated concentration of ZnCl_2_ or CdCl_2_ and incubated 16 hours at 37°C. Induced BbZIP (WT I) served as the positive control, whereas both uninduced BbZIP (WT UI) and empty vector [pET15b(+), EV] were included as negative controls. Assayed alanine substitutions cover D305 and S106 of the extracellular vestibule, E276, H275, and D144 of the intracellular bowl and M99 and H177 in the bundle-bundle interface. See Fig. 3 (A and B) for location of the mutated residues in BbZIP. (**B**) Immunoblot analysis of the corresponding crude lysates prepared from cells used in the metal toxicity assay. The tested alanine forms were produced at comparable levels as WT BbZIP. To detect BbZIP, an anti–His_6_-tag antibody conjugated with horseradish peroxidase was exploited. Representative results from a single experiment repeated three times are shown in both panels. Replicates are shown in fig. S11.

To verify the functional importance of these residues, we designed an in vivo metal toxicity assay, attributing decreased cell viability to increased zinc uptake. Cells with wild-type BbZIP were more intolerant to high Zn^2+^ or Cd^2+^ concentrations than control cells lacking the protein (Fig. 6A and fig. S11). To ensure an unbiased comparison of the mutant forms, the BbZIP protein levels in the cell membranes were also monitored (Fig. 6B). On the extracellular side, alanine substitution of S106 supports a critical role of this amino acid residue for the transport function, while the D305A form indicates that the residue is too peripheral to have a critical impact (Fig. 6A and fig. S11). Similarly, a series of functionally important electronegatively charged residues have been identified in this region of hZIP2 (i.e., H63, E67, E70, and E71), and mutation of the corresponding residue of S106 in hZIP4 (i.e., H379) severely diminished zinc transport (*16*). Intracellularly, changes of D144 and E276 to alanine indicate they play key roles in zinc transport, while H275A exhibited marginal effect on the function. Likewise, alanine replacements of M99 and H177 located between the scaffold and transport domains support the notion of this surface being tolerant to certain amino acid substitutions, as also previously shown for other elevator proteins (*31*, *32*).

### The elevator mechanism is compatible with the different transport modes of ZIPs

We note that differences in the setup of the metal-binding region may well explain the various transport modes observed for ZIP proteins, which overall are grouped into the four established subclasses, i.e., GufA, ZIPII, LIV-1, and ZIPI (Fig. 5 and fig. S12). Specifically, the stoichiometry per transport cycle varies among ZIPs. This correlates with a variable number of Zn^2+^ ions that bind to an omnipresent primary metal-binding site (M1) and an occasional second site (M2) (*12*, *17*). This trend is coupled to a corresponding diversity in the ligand configuration to the transported cargo. As an example, hZIP2 of subclass ZIPII, a passive pH-dependent transporter, has a lysine occupying the analog of M2 (fig. S10) and may hence directly modulate stoichiometry. Moreover, protonation of this lysine is essential for transport (*12*). As the protonation state is dictated by pH, extracellular pH likely regulates whether the metal-binding area during import is similarly charged as in GufA ZIPs such as BbZIP with two metal binding sites. Correspondingly, symport of HCO_3_^−^ as observed in hZIP4 and H^+^ as detected in hZIP8 may influence how ZIPs that cotransport other ions than Zn^2+^ contribute to electrical differences across membranes. For example, BbZIP has been shown to be electrogenic, an effect that may be reduced or enhanced by HCO_3_^−^ and H^+^, respectively. Moreover, it has been shown for other elevator-like transporter classes that the transport domain requires to be appropriately charge compensated to permit the turnover and to prevent slippage of cargo (fig. S12C) (*33*–*35*). The abovementioned ZIP transport modes likely affect the local charge of the transport domain as well.

Last, in recent studies, the classical definition of channels and transporters has been challenged, as elevator proteins with considerable conformational changes are able to transport cargo at relatively high rates. This indicates that stochastic processes are sufficient to drive large conformational changes and to achieve both fast and slow transport rates (*36*, *37*). This agrees with the observed channel-like kinetics of the transport rate of BbZIP, seemingly not rate-limited by conformational changes (*13*). Similar patterns with a large scope of transport modes and uncoupled nonsaturated transport characteristics have also been observed for the SLC26 family of anion elevator transporters (*38*). Ultimately, this suggests that nonsaturated ion passage across cellular membranes can be achieved not only by channels but also by elevator-like transporters such as ZIPs. Consequently, the elevator mechanism may well be compatible with the many different transport modes detected for ZIPs due to subtle local adaptations of the ion-binding region, altering coupling principle and modulating selectivity.

### The metal-free structure reveals an additional unique transmembrane segment of BbZIP, TMa

**Fig. 7.**
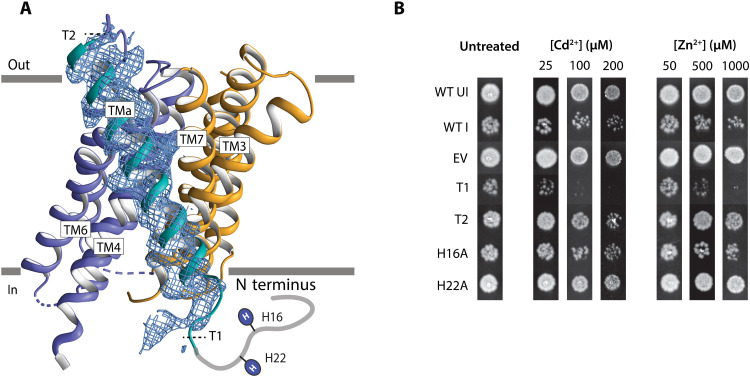
The additional unique transmembrane segment of BbZIP, TMa. (**A**) Side view of the inward-open metal-free structure with the final 2*F*_o_ − *F*_c_ electron density for the identified additional TM segment a, TMa, contoured at σ = 1.0 (blue mesh). (**B**) Assessment of the functional importance of the cytoplasmic N-terminal segment preceding TMa using the in vivo metal toxicity assay. *E. coli* cells [strain C43(DE3)] carrying wild-type BbZIP (WT), single mutations, or truncations of the N terminus were spotted on LB agar plates containing the indicated concentration of ZnCl_2_ or CdCl_2_ and incubated for 16 hours at 37°C. Induced BbZIP (WT I) served as the positive control, whereas both uninduced BbZIP (WT UI) and empty vector [pET15b(+), EV] were included as negative controls. Assayed variants include two N-terminal truncations, i.e., T1 (Δ3 to Δ20) and T2 (Δ3 to Δ52), and two single mutants, i.e., H16A and H22A. Representative results from a single experiment repeated three times are shown. Replicates are shown in fig. S11. Immunoblots showing the expression level of WT and mutants are presented in fig. S14.

**Fig. 8.**
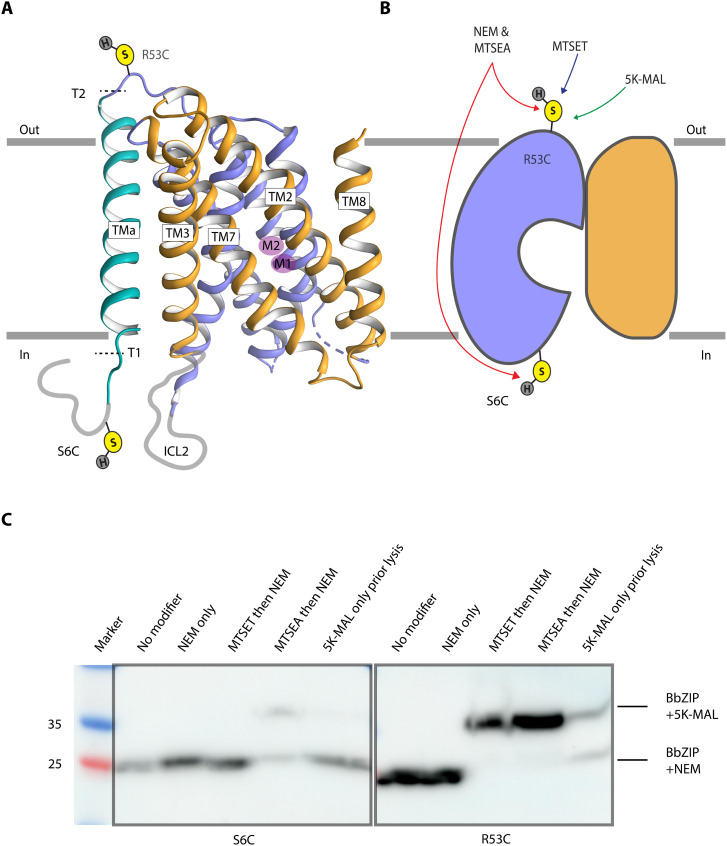
The cysteine accessibility assay. (**A**) BbZIP topology and placement of single-cysteine residues introduced to assess the location of TMa. (**B**) Overview of the cysteine accessibility assay. To assess the topology TMa, two separate single mutants introducing a cysteine residue on each side of TMa were generated: SC6 inside and R53C outside. MTSET (membrane impermeable) or MTSEA (permeable) was added before treatment with *N*-ethylmaleimide (NEM). NEM blocked cysteines accessible, not bound by MTSET or MTSEA. Reversible disulfide bonds of MTSET or MTSEA were removed by 1,4-dithiothreitol (DTT). Conversely, NEM reacts with exposed cysteine residues in an irreversible manner (if not protected by MTSET or MTSEA). Hence, only cysteines located on the extracellular side will react with MTSET, while all accessible cysteines will interact with MTSEA. Following exposure to DTT, accessible cysteines will react with 5K-MAL (5000 methoxypolyethylene glycol maleimide), resulting in increase of the total molecular weight. (**C**) Immunoblot analysis of S6C and R53C BbZIP mutants in the cysteine accessibility assay. Lanes 1 and 6 were untreated. Lanes 2 and 7 were treated with NEM and 5K-MAL. Lanes 3 and 8 were exposed to MTSET, NEM, and 5K-MAL. Lanes 4 and 9 were exposed to MTSEA, NEM, and 5K-MAL, while lanes 5 and 10 were treated only with 5K-MAL. Results show an up-shift of the protein band for the R53C mutant when treated with both MTSET and MTSEA, while S6C only experience an up-shift when treated with MTSEA. Thus, S6C is located intracellularly in vivo. For further details and a replicate, see fig. S13.

The additional transmembrane segment in BbZIP preceding TM1, TMa, is unexpected, not being visible in the previously determined structures. The resolved part represents 30 N-terminal amino acids and a highly tilted helix, interacting only with TM3 and TM6 and peripherally relative to the dimer assembly (Figs. 2B, 7, and 8A). As only approximately 20 residues are left unresolved, this feature is likely transforming previously proposed topology of the termini from an out/out to an in/out arrangement, with the N-terminal segment facing rather the intracellular side. To provide evidence that BbZIP exists with an in/out topology in vivo, we exploited a cysteine accessibility assay based on *Escherichia coli* cells expressing two different BbZIP forms, i.e., S6C in the N terminus or R53C following TMa (Figs. 7A and 8, A to C, and fig. S13) (*39*). When treating with the membrane-impermeable (2-[trimethylammonium]ethyl)methan-ethiosulfonate bromide (MTSET), only the R53C mutant displayed the electrophoretic shift associated with periplasmic localization, while the S6C form remained unaltered. However, when treating with the membrane-permeable 2-aminoethyl methanethiosulfonate hydrobromide (MTSEA), both S6C and R53C displayed an electrophoretic shift in agreement with S6C having a cytoplasmic localization. Thus, the in/out topology is present in vivo and does not reflect a crystallization artifact. On the basis of the metal-bound structures, it cannot be excluded that the position of the helix in the membrane is dynamic (snorkeling). However, it is more likely that this additional segment is simply flexible (perhaps detergent-induced) with maintained membrane position and, therefore, not visible in the earlier structures. It is tempting to speculate that many of the established difficulties in isolating ZIPs relate to the typical out/out topology, an arrangement known to be associated with complications with protein expression (*40*). In contrast, it is possible to recover acceptable quantities of BbZIP that exhibits an in/out setup. This may also hint at a general possible role of this additional helix in ZIPs beyond BbZIP, as a driver of expression to increase the accumulation of protein in the bacterial membranes, thereby increasing cellular Zn^2+^ import.

### The N terminus may modulate Zn^2+^ import among ZIPs with nine TMs

To further investigate the biological role of TMa and the N-terminal tail, we assessed additional BbZIP forms using the in vivo metal toxicity assay. Notably, we observed that a truncation T1, lacking the first 20 residues (∆3 to ∆20), appears hyperactive compared to the wild type. This suggests that the cytoplasmic N-terminal segment may be autoinhibitory or down-regulatory of Zn^2+^ transport (Fig. 7B and figs. S11 and S14). From a structural point of view, this is certainly possible, as the beginning of TMa is immediately adjacent to the ion release cavity (Fig. 8A). Moreover, it is located in the proximity of conserved features of the second intracellular loop (ICL2), a stretch that has been shown to be important for Zn^2+^ transport in hZIP4 (*41*). Thus, the tail may well interfere with metal release to the intracellular side. Conversely, both a more prominent truncation, removing all the residues before TM1, i.e., T2 (∆3 to ∆52), and the H22A mutation, interfering with a putative Zn^2+^-binding histidine present before TMa, decrease functionality. This agrees with the notion that H22 may partake in Zn^2+^ release. Conversely, the H16A variant of another histidine in the N terminus only moderately alters the activity. Thus, while not omnipresent among ZIPs, our data suggest that the identified N-terminal tail-TMa feature may be directly involved in Zn^2+^ transport and regulation in ZIPs with nine-TM topology. Mechanistically, it is hence possible that the N terminus of nine-TM ZIPs structurally and functionally replaces the distinctly longer and histidine-rich ICL2 of the human members. Noteworthy, all human ZIPs are predicted to share an eight-TM topology, and hence, they lack an intracellular tail, but the ICL2 instead contributes to zinc binding, thereby regulating Zn^2+^ transport activity (*42*, *43*).

### Summary

Fold and mechanism represent fundamental aspects in the understanding of protein function, providing insights valuable for both basic and translational sciences. In this work, we unraveled through structural, functional, and in silico analyses that the ZIP proteins likely operate as two-domain elevator-type transporters. Our results provide an extended conformational landscape for ZIP transporters. Furthermore, they highlight that this transporter family harbors an immobile dimer-forming scaffold domain and a transport domain that displaces a central ion-binding region to transfer cargo across membranes. The transport relies on symmetrically related negatively charged invaginations of the rigid scaffold domain shared within the dimer, limiting the displacement distance across the membrane required for passage. Moreover, the elevator transport mechanism and charge compensation principle can unify the functional and structural data available for ZIPs, with several different detected modes for ion transfer and selectivity. These are ranging from channel- to transporter-like models and from coupled to uncoupled passage, as achieved by subtle local differences around the binding region. Hence, elevator-type proteins appear rather diverse, as also previously observed, for example, in the CLC family of proteins that operate both as channels and as transporters despite overall very similar folds (*44*). We also demonstrate that certain ZIPs have an unorthodox topology, with an additional transmembrane segment in the N terminus. This extra helix may affect both the available cellular levels of the protein and its function. Undoubtedly, the structural and mechanistic insights into ZIPs and related elevator proteins will continue to grow. To fully understand the transport mechanism, additional structures, especially outward-facing conformations, are essential because of the shortage of architectural data. Concomitantly, more in-depth studies, such as cross-linking experiments with selected cysteine mutations or using linkers of variable lengths, have the potential to further validate and refine the ZIP transporter elevator mechanism revealed here. All in all, our results will prime these downstream efforts and provide a conceptual framework for analyses at the molecular level of the many known ZIP-related disorders.

## MATERIALS AND METHODS

### Overproduction and purification of BbZIP

The pET15b(+) vector encoding wild-type ZIP from *B. bronchiseptica* (BbZIP) (UniProt ID: A0A0H3LM39) codon-optimized for expression in *E. coli* and fused N-terminally with His_6_-tag, followed by the thrombin cleavage site was purchased from GenScript, USA. BbZIP mutants were prepared using the QuickChange Lightning Site-Directed Mutagenesis Kit (Agilent, USA) according to the instructions of the manufacturer. Primers used for engineering of the mutants were purchased from TAG Copenhagen A/S, Denmark, and their sequences are shown in table S2. Identity of the mutants was verified by DNA sequencing.

Precultures of *E. coli* [strain C43(DE3)] (Thermo Fisher Scientific, USA) were inoculated from frozen stocks and grown for 16 hours at 37°C in 5 ml of Luria-Bertani (LB) medium supplemented with ampicillin (100 μg/ml). Subsequently, 1 ml of the preculture was transferred into 50 ml of LB medium (containing ampicillin) and grown for ~1 hour at 37°C before the entire bacterial culture was used to inoculate 2 liters of Terrific broth medium (containing ampicillin). The resulting culture was grown at 37°C until optical density at 600 nm (OD_600_) = 0.9 before the protein expression was induced by adding isopropyl-β-d-thiogalactopyranoside to the final concentration of 0.5 mM. Following the induction, the expression was allowed for ~18 hours at 20°C before the cells were harvested by centrifugation at 6000*g* for 10 min at 4°C. Obtained cell pellets were resuspended with ice-cold lysis buffer [50 mM Hepes-NaOH (pH 7.3), 500 mM NaCl, and 20% glycerol] and kept at −80°C until use. Thawed cell pellets were supplemented with cOmplete protease inhibitor cocktail (Sigma-Aldrich, USA) and opened by purging twice through a cell disrupter (Constant Systems Limited, UK) operated at 26 kpsi. After cell disruption, 1 mM phenylmethylsulfonyl fluoride was added, and the cell lysate was centrifuged at 20,000*g* for 20 min at 4°C to remove cell debris. Subsequently, the membrane fraction was isolated by ultracentrifugation using Ti45 rotor (Beckman, USA) at 138,000*g* for 3 hours at 4°C. Obtained crude membranes were resuspended in solubilization buffer [10 ml/g; 50 mM Hepes-NaOH (pH 7.3), 200 mM NaCl, and 20% glycerol] and kept at −80°C until use.

### Purification of BbZIP for crystallization

Membranes at the total protein concentration of 5 mg/ml were solubilized with 1.5 to 2% (w/v) *n*-dodecyl-β-d-maltopyranoside (DDM) (Glycon Biochemicals, Germany) for 4 hours in the presence of the 0.1 mM CdCl_2_. Subsequently, solubilized membranes were diluted to reduce concentration of DDM to 0.33% (w/v), and the additional NaCl was added to increase the concentration to 500 mM before the material was loaded on a 5 ml of HisTrap HP column (Cytiva, Denmark). Bound protein was washed with immobilized-metal affinity chromatography (IMAC) buffer [50 mM Hepes-NaOH (pH 7.3), 200 mM NaCl, 20% glycerol, 50 mM imidazole, and 0.1% (w/v) DDM] and eluted in IMAC buffer using a linear imidazole gradient (50 to 500 mM). Fractions containing protein were pooled and immediately dialyzed against IMAC buffer for 16 hours at 4°C. Subsequently, protein was concentrated in a Vivaspin 20 centrifugal concentrator (MWCO, 50 kDa; Sartorius, Germany) to ~4 mg/ml. The sample was loaded onto a Superdex Increase 200 10/300 GL column (Cytiva) equilibrated with size exclusion chromatography (SEC) buffer [20 mM NaOAc-HOAc (pH 4.5), 200 mM NaCl, 15% glycerol, and 0.03% (w/v) DDM].

### Crystallization of BbZIP

SEC-pure BbZIP was concentrated to 16 to 36 mg/ml as described above and filtered, and 10 to 20 μl of the sample was loaded into a 100-μl glass syringe (Hamilton, USA). Subsequently, the sample was mixed with ~1.5× volume of molten monoolein (Sigma-Aldrich, USA) to reach the weight ratio of 3:2 (lipid:protein) using the two-syringe method with engaged coupler. Both solutions were purged through the coupler ~100 times until a transparent LCP was achieved. The resulting mixture was then transferred to a dispensing syringe and mounted on the Crystal Gryphon LCP robot (Art Robbins Instruments, USA) that dispensed 35-, 50-, or 100-nl bolus drops on 96-well custom glass plates. Subsequently, the drops were overlaid with 800 nl of precipitant solution and incubated onward at 20°C.

Crystals of wild-type metal-free BbZIP (achieved for the protein concentration of 36 mg/ml) appeared after 2 weeks in 100 mM NaOAc-HOAc (pH 4.5), 100 to 400 mM LiSO_4_, and 25 to 40% PEG400 (polyethylene glycol, molecular weight 400). The best diffracting crystals were obtained in 300 mM LiSO_4_ and 35% PEG400 and grew to a size of 10 to 30 μm with the shape of trapezoidal prisms. Crystals of Cd^2+^-bound BbZIP appeared after 10 days in 100 mM Hepes-NaOH (pH 7.4), 100 mM CdCl_2_, and 30% PEG400 and grew to full size after 10 days. Crystals were allowed to grow for additional 2 weeks before being fished into 0.06-mm loops (Molecular Dimensions Limited, UK) and flash-frozen in liquid nitrogen.

### Data collection and structure determination

Diffraction data were collected at the beamline PXIII X06SA (Swiss Light Source, Switzerland). The data were indexed, integrated, and scaled using the XDS package (*45*). The crystal structures of BbZIP were determined by molecular replacement in PHASER (*46*) using the metal-bound BbZIP structure [Protein Data Bank (PDB) ID: 5TSA] (*16*) as the search model. COOT (*47*) was used for model building, and refinement was performed in PHENIX (*48*). Assignment of registry for the TMa and further rebuilding of the model was performed using ISOLDE (*49*), followed by final refinement in PHENIX. Information about data collection and refinement statistics is shown in table S1. Metal-free BbZIP contains two molecules in the asymmetric unit. Electron densities were observed for residues 21 to 145, 165 to 279, and 285 to 309 in chain A and for residues 18 to 77, 84 to 145, 166 to 219, and 225 to 307 in chain B, respectively. Chain B was used for further analysis and generation of figures.

### RMSD calculations and distance difference matrices

Calculations of RMSD values of residues were performed with the ColorByRMSD PyMOL script. Calculations of RMSD values of structures were performed with the super command in PyMOL. Distance difference matrices and plots were created with a previously reported Python-based script (https://github.com/GaudetLab/archived-DDMP/) (*50*). The TM definitions from the metal-bound BbZIP structure (PDB ID: 5TSA) (*16*) were used for the calculations.

### Analysis of evolutionary coupled amino acid residues

The sequence of wild-type BbZIP was used as a query. Automatic identification of the sequences annotated with the Pfam PF02535 ZIP identifier was used to generate the multiple sequence alignment (MSA) used as input for the sequence coevolution analysis using the EVCS implementing the plmDCA algorithm (https://evcouplings2.hms.harvard.edu/) (*19*). Analysis was performed with default parameters, and 22,433 effective sequences were exploited for analysis with the overall high effective sequence-to-protein length ratio (i.e., 90.1 seq/liter). The N-terminal region of BbZIP was not covered in the alignment. Significance cutoff was set to 0.9 in the subsequent plot visualizations.

### Generation of the outward-facing model of BbZIP

The outward-facing model of BbZIP was generated on the basis of serial translations of the transport domain of the inward-open metal-bound structure across the membrane toward the outside. The intention was to minimize the distance between evolutionary coupled residues in-between the two helix bundles that were unsatisfied in the inward-facing conformation, namely, Ser^106^-Gln^207^, Ser^261^-Met^183^, Val^272^-Thr^173^, Ala^102^-Gln^207^, Met^269^-Glu^211^, and Met^269^-Asp^208^. To minimize the distance between these residue pairs, a stepwise translation across the membrane in steps of 1 Å was performed. The shortest total distance between the residues was obtained through an 8 Å translation, thereby satisfying the abovementioned evolutionary coupled pairs.

### Cysteine accessibility assay

Cell pellets resulting from cultures of *E. coli* [strain C43(DE3)] expressing single-cysteine BbZIP mutants were washed one time in labeling buffer [100 mM tris-HCl (pH 7), 60 mM NaCl, and 10 mM KCl] and subsequently resuspended in labeling buffer to the OD_600_ = 4. Subsequently, 100 μl of the cell suspension was aliquoted per well in a 96-well plate and mixed with either 100 μl of 6 mM MTSET (Biotium, USA) or 100 μl of 6 mM MTSEA (Biotium, USA). The mixture was incubated for 30 min before the addition of 5 μl of 60 mM *N*-ethylmaleimide (NEM; Sigma-Aldrich, USA) and incubated for additional 30 min. Subsequently, 10 μl of 200 mM l-cysteine was applied for 15 min to quench-free MTSET, MTSEA, and NEM. After two consecutive washes in labeling buffer, cells were resuspended in 60 μl of denaturing buffer (100 mM tris-HCl (pH 7), 6 M urea, 0.5% SDS, and 0.5 mM 1,4-dithiothreitol) and incubated for 1 hour. Samples were then centrifuged at 2700*g* for 15 min at 18°C. Subsequently, 20 μl of the supernatant was mixed with 7 μl of 6 mM 5000 methoxypolyethylene glycol maleimide (Sigma-Aldrich, USA) and incubated for 1 hour at 37°C before 5.4 μl of 5× SDS sample buffer containing 700 mM β-mercaptoethanol was added to stop the reaction. As a negative control, 100 μl of the labeling buffer was applied instead of the thiol modifiers. Subsequently, 25 μl of the respective samples were resolved on 4 to 20% SDS–polyacrylamide gel electrophoresis gels (Thermo Fisher Scientific). Protein bands were then detected using immunoblotting, using an anti-His_6_ monoclonal antibody–horseradish peroxidase conjugate (Takara Bio, USA) according to recommendations of the manufacturer. Immunoblots were visualized by chemiluminescence with SuperSignal West Femto Maximum Sensitivity substrate (Thermo Fisher Scientific) using an AlphaImager (Alpha Innotech, USA) machine. Relative signal intensities of the bands were quantified using ImageJ software (https://imagej.nih.gov/ij/) (*51*). Each experiment was conducted using two biological replicates.

### In vivo metal toxicity assay

*E. coli* [strain C43(DE3)] cultures were allowed to express wild-type BbZIP and the respective mutants for 4 hours at 37°C before being spotted (1 μl at OD_600_ = 0.001) on LB agar plates containing the corresponding concentrations of metal. Plates were then incubated for 16 hours at 37°C before the colonies were imaged using an AlphaImager (Alpha Innotech) scanner. Contrast between cell colonies and the background agar was increased by systematically readjusting the gamma values in the ImageJ software (*51*). Each experiment was conducted using three biological replicates.

### Protein-protein docking

Possible dimerization sites of BbZIP were evaluated by a protein-protein docking strategy. Identical monomeric chains were docked blindly into all possible interfaces to the monomer itself. Monomers were allowed to rotate 360° while keeping the central protein monomer rigid. The docking was performed using the protein-protein docking module in the MOE-2016.0802 software (*52*). The affinity binding energies for 100 dimers were calculated on the basis of Amber94 force field parameters (*53*) and were ranked according to the binding score from lowest to the highest. The affinity binding score or docking energy was estimated from the enthalpic contribution to the binding free energy between two binding monomers. In addition, each docking pose was refined and resulted in refinement energy. The docking poses were clustered on the basis of the docking and refinement energy parameters by the prediction analysis of microarrays method (*21*) and using the Clusplot function in the R software to identify related clusters based on their energy values (*54*).

## Supplementary Materials

### This PDF file includes:

Figs. S1 to S14 Tables S1 and S2 References

## Notes

View/request a protocol for this paper from *Bio-protocol*.

## References

[1] T. Hara, T.-A. Takeda, T. Takagishi, K. Fukue, T. Kambe, T. Fukada, Physiological roles of zinc transporters: Molecular and genetic importance in zinc homeostasis. J. Physiol. Sci. 67, 283–301 (2017). 28130681 10.1007/s12576-017-0521-4 PMC10717645

[2] C. Andreini, L. Banci, I. Bertini, A. Rosato, Counting the zinc-proteins encoded in the human genome. J. Proteome Res. 5, 196–201 (2006). 16396512 10.1021/pr050361j

[3] B. H. Bin, J. Seo, S. T. Kim, Function, structure, and transport aspects of ZIP and ZnT Zinc transporters in immune cells. J. Immunol. Res. 2018, 9365747 (2018). 30370308 10.1155/2018/9365747 PMC6189677

[4] S. Küry, B. Dréno, S. Bézieau, S. Giraudet, M. Kharfi, R. Kamoun, J. P. Moisan, Identification of SLC39A4, a gene involved in acrodermatitis enteropathica. Nat. Genet. 31, 239–240 (2002). 12068297 10.1038/ng913

[5] T. Fukada, N. Civic, T. Furuichi, S. Shimoda, K. Mishima, H. Higashiyama, Y. Idaira, Y. Asada, H. Kitamura, S. Yamasaki, S. Hojyo, M. Nakayama, O. Ohara, H. Koseki, H. G. dos Santos, L. Bonafe, R. Ha-Vinh, A. Zankl, S. Unger, M. E. Kraenzlin, J. S. Beckmann, I. Saito, C. Rivolta, S. Ikegawa, A. Superti-Furga, T. Hirano, The zinc transporter SLC39A13/ZIP13 is required for connective tissue development; its involvement in BMP/TGF-β signaling pathways. PLOS ONE 3, e3642 (2008). 18985159 10.1371/journal.pone.0003642 PMC2575416

[6] L. A. Gaither, D. J. Eide, Functional expression of the human hZIP2 zinc transporter. J. Biol. Chem. 275, 5560–5564 (2000). 10681536 10.1074/jbc.275.8.5560

[7] L. He, K. Girijashanker, T. P. Dalton, J. Reed, H. Li, M. Soleimani, D. W. Nebert, ZIP8, member of the solute-carrier-39 (SLC39) metal-transporter family: Characterization of transporter properties. Mol. Pharmacol. 70, 171–180 (2006). 16638970 10.1124/mol.106.024521

[8] K. Girijashanker, L. He, M. Soleimani, J. M. Reed, H. Li, Z. Liu, B. Wang, T. P. Dalton, D. W. Nebert, Slc39a14 gene encodes ZIP14, a metal/bicarbonate symporter: Similarities to the ZIP8 transporter. Mol. Pharmacol. 73, 1413–1423 (2008). 18270315 10.1124/mol.107.043588 PMC2753210

[9] L. A. Gaither, D. J. Eide, The human ZIP1 transporter mediates zinc uptake in human K562 erythroleukemia cells. J. Biol. Chem. 276, 22258–22264 (2001). 11301334 10.1074/jbc.M101772200

[10] Z. Liu, H. Li, M. Soleimani, K. Girijashanker, J. M. Reed, L. He, T. P. Dalton, D. W. Nebert, Cd^2+^ versus Zn^2+^ uptake by the ZIP8 HCO_3_^−^-dependent symporter: Kinetics, electrogenicity and trafficking. Biochem. Biophys. Res. Commun. 365, 814–820 (2008). 18037372 10.1016/j.bbrc.2007.11.067 PMC2212618

[11] E. Hoch, M. Levy, M. Hershfinkel, I. Sekler, Elucidating the H^+^ coupled Zn^2+^ transport mechanism of ZIP4; implications in acrodermatitis enteropathica. Int. J. Mol. Sci. 21, 734 (2020). 31979155 10.3390/ijms21030734 PMC7037870

[12] G. Gyimesi, G. Albano, D. G. Fuster, M. A. Hediger, J. Pujol-Giménez, Unraveling the structural elements of pH sensitivity and substrate binding in the human zinc transporter SLC39A2 (ZIP2). J. Biol. Chem. 294, 8046–8063 (2019). 30914478 10.1074/jbc.RA118.006113 PMC6527156

[13] W. Lin, J. Chai, J. Love, D. Fu, Selective electrodiffusion of zinc ions in a Zrt-, Irt-like protein, ZIPB. J. Biol. Chem. 285, 39013–39020 (2010). 20876577 10.1074/jbc.M110.180620 PMC2998139

[14] B. H. Bin, T. Fukada, T. Hosaka, S. Yamasaki, W. Ohashi, S. Hojyo, T. Miyai, K. Nishida, S. Yokoyama, T. Hirano, Biochemical characterization of human ZIP13 protein: A homo-dimerized zinc transporter involved in the spondylocheiro dysplastic Ehlers-Danlos syndrome. J. Biol. Chem. 286, 40255–40265 (2011). 21917916 10.1074/jbc.M111.256784 PMC3220551

[15] T. P. Ajeesh Krishna, T. Maharajan, G. Victor Roch, S. Ignacimuthu, S. Antony Ceasar, Structure, function, regulation and phylogenetic relationship of ZIP family transporters of plants. Front. Plant. Sci. 11, 662 (2020). 32536933 10.3389/fpls.2020.00662 PMC7267038

[16] T. Zhang, J. Liu, M. Fellner, C. Zhang, D. Sui, J. Hu, Crystal structures of a ZIP zinc transporter reveal a binuclear metal center in the transport pathway. Sci. Adv. 3, e1700344 (2017). 28875161 10.1126/sciadv.1700344 PMC5573306

[17] T. Zhang, D. Sui, C. Zhang, L. Cole, J. Hu, Asymmetric functions of a binuclear metal center within the transport pathway of a human zinc transporter ZIP4. FASEB J. 34, 237–247 (2020). 31914589 10.1096/fj.201902043R PMC6956730

[18] T. Zhang, D. Sui, J. Hu, Structural insights of ZIP4 extracellular domain critical for optimal zinc transport. Nat. Commun. 7, 11979 (2016). 27321477 10.1038/ncomms11979 PMC4915132

[19] T. A. Hopf, A. G. Green, B. Schubert, S. Mersmann, C. P. I. Schärfe, J. B. Ingraham, A. Toth-Petroczy, K. Brock, A. J. Riesselman, P. Palmedo, C. Kang, R. Sheridan, E. J. Draizen, C. Dallago, C. Sander, D. S. Marks, The EVcouplings Python framework for coevolutionary sequence analysis. Bioinformatics 35, 1582–1584 (2019). 30304492 10.1093/bioinformatics/bty862 PMC6499242

[20] M. Mirdita, S. Ovchinnikov, M. Steinegger, ColabFold—Making protein folding accessible to all. bioRxiv 10.1101/2021.08.15.456425 [**Preprint**]. 2021. 10.1038/s41592-022-01488-1 PMC9184281 35637307

[21] R. Tibshirani, T. Hastie, B. Narasimhan, G. Chu, Diagnosis of multiple cancer types by shrunken centroids of gene expression. Proc. Natl. Acad. Sci. U.S.A. 99, 6567–6572 (2002). 12011421 10.1073/pnas.082099299 PMC124443

[22] S. Antala, S. Ovchinnikov, H. Kamisetty, D. Baker, R. E. Dempski, Computation and functional studies provide a model for the structure of the zinc transporter hZIP4. J. Biol. Chem. 290, 17796–17805 (2015). 25971965 10.1074/jbc.M114.617613 PMC4505028

[23] F. M. Richards, C. E. Kundrot, Identification of structural motifs from protein coordinate data: Secondary structure and first-level supersecondary structure. Proteins 3, 71–84 (1988). 3399495 10.1002/prot.340030202

[24] A. A. Garaeva, D. J. Slotboom, Elevator-type mechanisms of membrane transport. Biochem. Soc. Trans. 48, 1227–1241 (2020). 32369548 10.1042/BST20200290 PMC7329351

[25] D. Drew, O. Boudker, Shared molecular mechanisms of membrane transporters. Annu. Rev. Biochem. 85, 543–572 (2016). 27023848 10.1146/annurev-biochem-060815-014520

[26] T. A. Hopf, L. J. Colwell, R. Sheridan, B. Rost, C. Sander, D. S. Marks, Three-dimensional structures of membrane proteins from genomic sequencing. Cell 149, 1607–1621 (2012). 22579045 10.1016/j.cell.2012.04.012 PMC3641781

[27] B. Byrne, It takes two to transport via an elevator. Cell Res. 27, 965–966 (2017). 28685771 10.1038/cr.2017.89 PMC5539353

[28] C. Lee, H. J. Kang, C. von Ballmoos, S. Newstead, P. Uzdavinys, D. L. Dotson, S. Iwata, O. Beckstein, A. D. Cameron, D. Drew, A two-domain elevator mechanism for sodium/proton antiport. Nature 501, 573–577 (2013). 23995679 10.1038/nature12484 PMC3914025

[29] C. Wang, B. Sun, X. Zhang, X. Huang, M. Zhang, H. Guo, X. Chen, F. Huang, T. Chen, H. Mi, F. Yu, L. N. Liu, P. Zhang, Structural mechanism of the active bicarbonate transporter from cyanobacteria. Nat. Plants 5, 1184–1193 (2019). 31712753 10.1038/s41477-019-0538-1

[30] B. H. Thurtle-Schmidt, R. M. Stroud, Structure of Bor1 supports an elevator transport mechanism for SLC4 anion exchangers. Proc. Natl. Acad. Sci. U.S.A. 113, 10542–10546 (2016). 27601653 10.1073/pnas.1612603113 PMC5035872

[31] M. Hirschi, Z. L. Johnson, S.-Y. Lee, Visualizing multistep elevator-like transitions of a nucleoside transporter. Nature 545, 66–70 (2017). 28424521 10.1038/nature22057 PMC5567992

[32] M. Duffield, A. Patel, O. V. Mortensen, D. Schnur, A. D. Gonzalez-Suarez, D. Torres-Salazar, A. C. K. Fontana, Transport rate of EAAT2 is regulated by amino acid located at the interface between the scaffolding and substrate transport domains. Neurochem. Int. 139, 104792 (2020). 32668264 10.1016/j.neuint.2020.104792

[33] D. B. Sauer, N. Trebesch, J. J. Marden, N. Cocco, J. Song, A. Koide, S. Koide, E. Tajkhorshid, D.-N. Wang, Structural basis for the reaction cycle of DASS dicarboxylate transporters. eLife 9, e61350 (2020). 32869741 10.7554/eLife.61350 PMC7553777

[34] J. S. Lolkema, D. J. Slotboom, Structure and elevator mechanism of the Na^+^-citrate transporter CitS. Curr. Opin. Struct. Biol. 45, 1–9 (2017). 27776291 10.1016/j.sbi.2016.10.004

[35] C. Grewer, Z. Zhang, J. Mwaura, T. Albers, A. Schwartz, A. Gameiro, Charge compensation mechanism of a Na^+^-coupled, secondary active glutamate transporter. J. Biol. Chem. 287, 26921–26931 (2012). 22707712 10.1074/jbc.M112.364059 PMC3411028

[36] P. Uzdavinys, M. Coinçon, E. Nji, M. Ndi, I. Winkelmann, C. Von Ballmoos, D. Drew, Dissecting the proton transport pathway in electrogenic Na^+^/H^+^ antiporters. Proc. Natl. Acad. Sci. U.S.A. 114, E1101–E1110 (2017). 28154142 10.1073/pnas.1614521114 PMC5321028

[37] E. Frumence, S. Genetet, P. Ripoche, A. Iolascon, I. Andolfo, C. L. Van Kim, Y. Colin, I. Mouro-Chanteloup, C. Lopez, Rapid CL^−^/HCO_3_^−^ exchange kinetics of AE1 in HEK293 cells and hereditary stomatocytosis red blood cells. Am. J. Physiol. Cell Physiol. 305, 654–662 (2013). 10.1152/ajpcell.00142.2013 23842529

[38] J. D. Walter, M. Sawicka, R. Dutzler, Cryo-EM structures and functional characterization of murine Slc26a9 reveal mechanism of uncoupled chloride transport. eLife 8, e46986 (2019). 31339488 10.7554/eLife.46986 PMC6656431

[39] A. T. Bozzi, L. B. Bane, W. A. Weihofen, A. Singharoy, E. R. Guillen, H. L. Ploegh, K. Schulten, R. Gaudet, Crystal structure and conformational change mechanism of a bacterial nramp-family divalent metal transporter. Structure 24, 2102–2114 (2016). 27839948 10.1016/j.str.2016.09.017 PMC5143219

[40] C. Ma, Z. Hao, G. Huysmans, A. Lesiuk, P. Bullough, Y. Wang, M. Bartlam, S. E. Phillips, J. D. Young, A. Goldman, S. A. Baldwin, V. L. G. Postis, A versatile strategy for production of membrane proteins with diverse topologies: Application to investigation of bacterial homologues of human divalent metal ion and nucleoside transporters. PLOS ONE 10, e0143010 (2015). 26606682 10.1371/journal.pone.0143010 PMC4659628

[41] S. Antala, R. E. Dempski, The human ZIP4 transporter has two distinct binding affinities and mediates transport of multiple transition metals. Biochemistry 51, 963–973 (2012). 22242765 10.1021/bi201553p

[42] K. M. Taylor, R. I. Nicholson, The LZT proteins; the LIV-1 subfamily of zinc transporters. Biochim. Biophys. Acta 1611, 16–30 (2003). 12659941 10.1016/s0005-2736(03)00048-8

[43] E. M. Bafaro, M. W. Maciejewski, J. C. Hoch, R. E. Dempski, Concomitant disorder and high-affinity zinc binding in the human zinc- and iron-regulated transport protein 4 intracellular loop. Protein Sci. 28, 868–880 (2019). 30793391 10.1002/pro.3591 PMC6459995

[44] E. Park, R. Mackinnon, Structure of the CLC-1 chloride channel from *Homo Sapiens*. eLife 7, e36629 (2018). 29809153 10.7554/eLife.36629 PMC6019066

[45] W. Kabsch, XDS. Acta Crystallogr. D Biol. Crystallogr. 66, 125–132 (2010). 20124692 10.1107/S0907444909047337 PMC2815665

[46] A. J. McCoy, R. W. Grosse-Kunstleve, P. D. Adams, M. D. Winn, L. C. Storoni, R. J. Read, Phaser crystallographic software. J. Appl. Cryst. 40, 658–674 (2007). 19461840 10.1107/S0021889807021206 PMC2483472

[47] P. Emsley, K. Cowtan, Coot: Model-building tools for molecular graphics. Acta Crystallogr. D Biol. Crystallogr. 60, 2126–2132 (2004). 15572765 10.1107/S0907444904019158

[48] P. D. Adams, P. V. Afonine, G. Bunkóczi, V. B. Chen, I. W. Davis, N. Echols, J. J. Headd, L. W. Hung, G. J. Kapral, R. W. Grosse-Kunstleve, A. J. McCoy, N. W. Moriarty, R. Oeffner, R. J. Read, D. C. Richardson, J. S. Richardson, T. C. Terwilliger, P. H. Zwart, PHENIX: A comprehensive Python-based system for macromolecular structure solution. Acta Crystallogr. D Biol. Crystallogr. 66, 213–221 (2010). 20124702 10.1107/S0907444909052925 PMC2815670

[49] T. I. Croll, ISOLDE: A physically realistic environment for model building into low-resolution electron-density maps. Acta Crystallogr. D Struct. Biol. 74, 519–530 (2018). 29872003 10.1107/S2059798318002425 PMC6096486

[50] A. T. Bozzi, C. M. Zimanyi, J. M. Nicoludis, B. K. Lee, C. H. Zhang, R. Gaudet, Structures in multiple conformations reveal distinct transition metal and proton pathways in an nramp transporter. eLife 8, e41124 (2019). 30714568 10.7554/eLife.41124 PMC6398981

[51] C. A. Schneider, W. S. Rasband, K. W. Eliceiri, NIH Image to ImageJ: 25 years of image analysis. Nat. Methods 9, 671–675 (2012). 22930834 10.1038/nmeth.2089 PMC5554542

[52] S. Vilar, G. Cozza, S. Moro, Medicinal chemistry and the molecular operating environment (MOE): Application of QSAR and molecular docking to drug discovery. Curr. Top. Med. Chem. 8, 1555–1572 (2008). 19075767 10.2174/156802608786786624

[53] C. I. Bayly, K. M. Merz, D. M. Ferguson, W. D. Cornell, T. Fox, J. W. Caldwell, P. A. Kollman, P. Cieplak, I. R. Gould, D. C. Spellmeyer, A second generation force field for the simulation of proteins, nucleic acids, and organic molecules. J. Am. Chem. Soc. 117, 5179–5197 (1995).

[54] R Core Team, *R: A Language and Environment for Statistical Computing* (R Foundation for Statistical Computing, 2011), vol. 1.

[55] H. Ashkenazy, S. Abadi, E. Martz, O. Chay, I. Mayrose, T. Pupko, N. Ben-Tal, ConSurf 2016: An improved methodology to estimate and visualize evolutionary conservation in macromolecules. Nucleic Acids Res. 44, W344–W350 (2016). 27166375 10.1093/nar/gkw408 PMC4987940

[56] J. Mistry, S. Chuguransky, L. Williams, M. Qureshi, G. A. Salazar, E. L. L. Sonnhammer, S. C. E. Tosatto, L. Paladin, S. Raj, L. J. Richardson, R. D. Finn, A. Bateman, Pfam: The protein families database in 2021. Nucleic Acids Res. 49, D412–D419 (2021). 33125078 10.1093/nar/gkaa913 PMC7779014

[57] K. D. Tsirigos, C. Peters, N. Shu, L. Käll, A. Elofsson, The TOPCONS web server for consensus prediction of membrane protein topology and signal peptides. Nucleic Acids Res. 43, W401–W407 (2015). 25969446 10.1093/nar/gkv485 PMC4489233

[58] J. D. Thompson, D. G. Higgins, T. J. Gibson, CLUSTAL W: Improving the sensitivity of progressive multiple sequence alignment through sequence weighting, position-specific gap penalties and weight matrix choice. Nucleic Acids Res. 22, 4673–4680 (1994). 7984417 10.1093/nar/22.22.4673 PMC308517

[59] I. Letunic, P. Bork, Interactive Tree of Life (iTOL) v4: Recent updates and new developments. Nucleic Acids Res. 47, W256–W259 (2019). 30931475 10.1093/nar/gkz239 PMC6602468

[60] E. Krissinel, K. Henrick, Inference of macromolecular assemblies from crystalline state. J. Mol. Biol. 372, 774–797 (2007). 17681537 10.1016/j.jmb.2007.05.022

[61] X. Yu, G. Yang, C. Yan, J. L. Baylon, J. Jiang, H. Fan, G. Lu, K. Hasegawa, H. Okumura, T. Wang, E. Tajkhorshid, S. Li, N. Yan, Dimeric structure of the uracil: Proton symporter UraA provides mechanistic insights into the SLC4/23/26 transporters. Cell Res. 27, 1020–1033 (2017). 28621327 10.1038/cr.2017.83 PMC5539350

[62] E. R. Geertsma, Y. N. Chang, F. R. Shaik, Y. Neldner, E. Pardon, J. Steyaert, R. Dutzler, Structure of a prokaryotic fumarate transporter reveals the architecture of the SLC26 family. Nat. Struct. Mol. Biol. 22, 803–808 (2015). 26367249 10.1038/nsmb.3091

[63] Y. Alguel, S. Amillis, J. Leung, G. Lambrinidis, S. Capaldi, N. J. Scull, G. Craven, S. Iwata, A. Armstrong, E. Mikros, G. Diallinas, A. D. Cameron, B. Byrne, Structure of eukaryotic purine/H^+^ symporter UapA suggests a role for homodimerization in transport activity. Nat. Commun. 7, 11336 (2016). 27088252 10.1038/ncomms11336 PMC4837479

[64] H. M. Berman, J. Westbrook, Z. Feng, G. Gilliland, T. N. Bhat, H. Weissig, I. N. Shindyalov, P. E. Bourne, The Protein Data Bank. Nucleic Acids Res. 28, 235–242 (2000). 10592235 10.1093/nar/28.1.235 PMC102472

