# Supplementary material for: The two-domain elevator-type mechanism of zinc-transporting ZIP proteins
Source: Sci Adv. 2022 Jul 13;8(28):eabn4331. doi: 10.1126/sciadv.abn4331 (PMC9278863; doi:10.1126/sciadv.abn4331)
Supplement: Supplementary file 1 — Figs. S1 to S14 Tables S1 and S2 References [file sciadv.abn4331_sm.pdf]

Science Advances
AAAS

Supplementary Materials for

The two-domain elevator-type mechanism of zinc-transporting ZIP proteins

Anders Wiuf *et al.*

Corresponding author: Kamil Gotfryd, kamil@sund.ku.dk; Pontus Gourdon, pontus@sund.ku.dk

Sci. Adv. **8**, eabn4331 (2022)
DOI: 10.1126/sciadv.abn4331

This PDF file includes:

Figs. S1 to S14
Tables S1 and S2
References

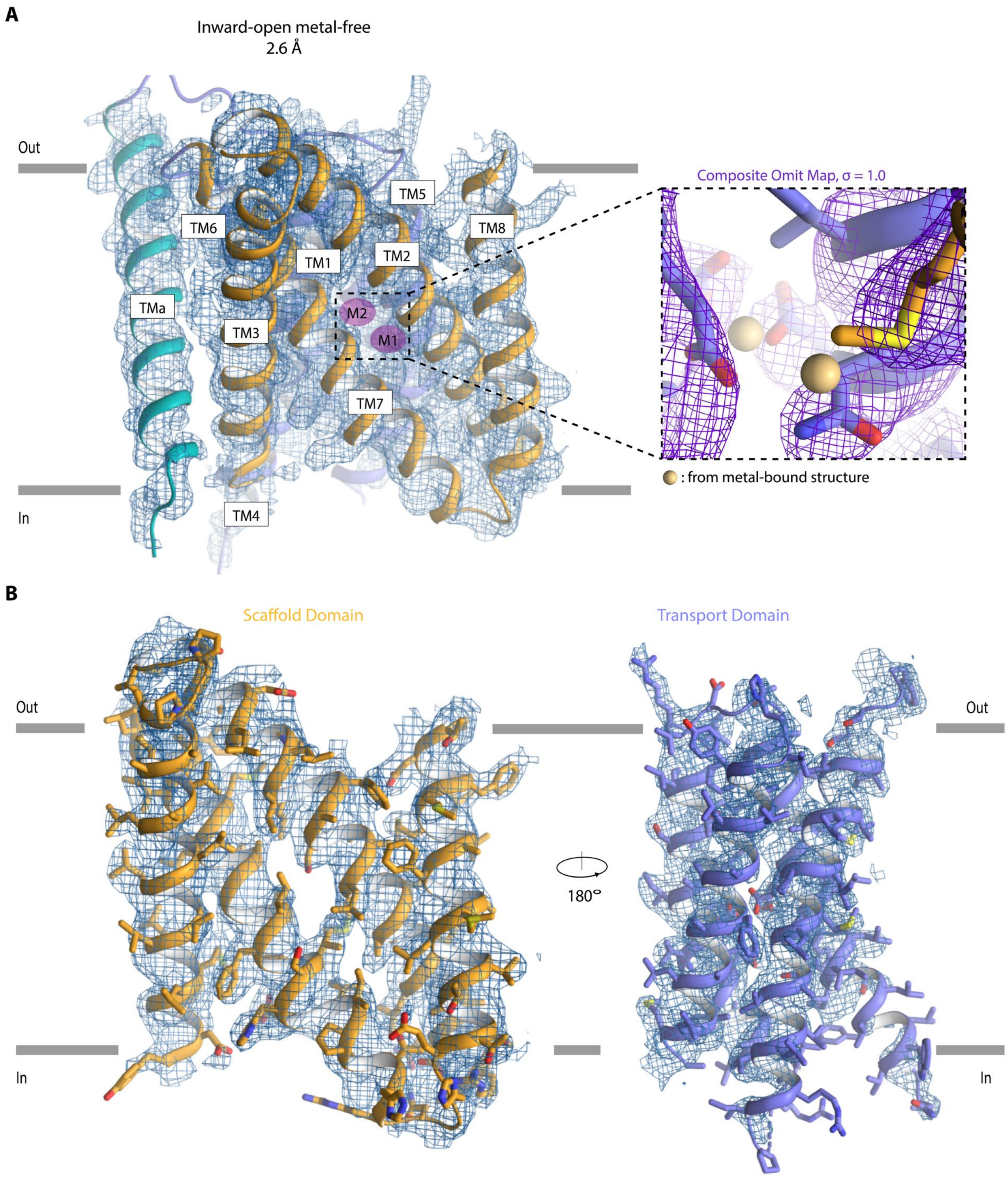

Fig. S1. **Electron density quality of the inward-open metal-free structure**. The final 2Fo-Fc electron density map contoured at σ=1.0 (blue mesh) if not stated otherwise. The overall resolution is indicated, and the structures are colored as in Fig. 1. **(A)** Overall structure of the inward-open metal-free shown as cartoon, the view is similar to Fig. 1. Purple spheres indicate the position of the metal-binding sites (M1 and M2) found in the metal-bound structure. Close-view: Unbiased composite omit map (purple mesh,

σ=1.0) of the metal-binding region indicates the area is void of density. **(B)** Left panel: the scaffold domain shown in the same view as in panel A. Right panel: the transport domain as observed from the opposite side (180° rotation).

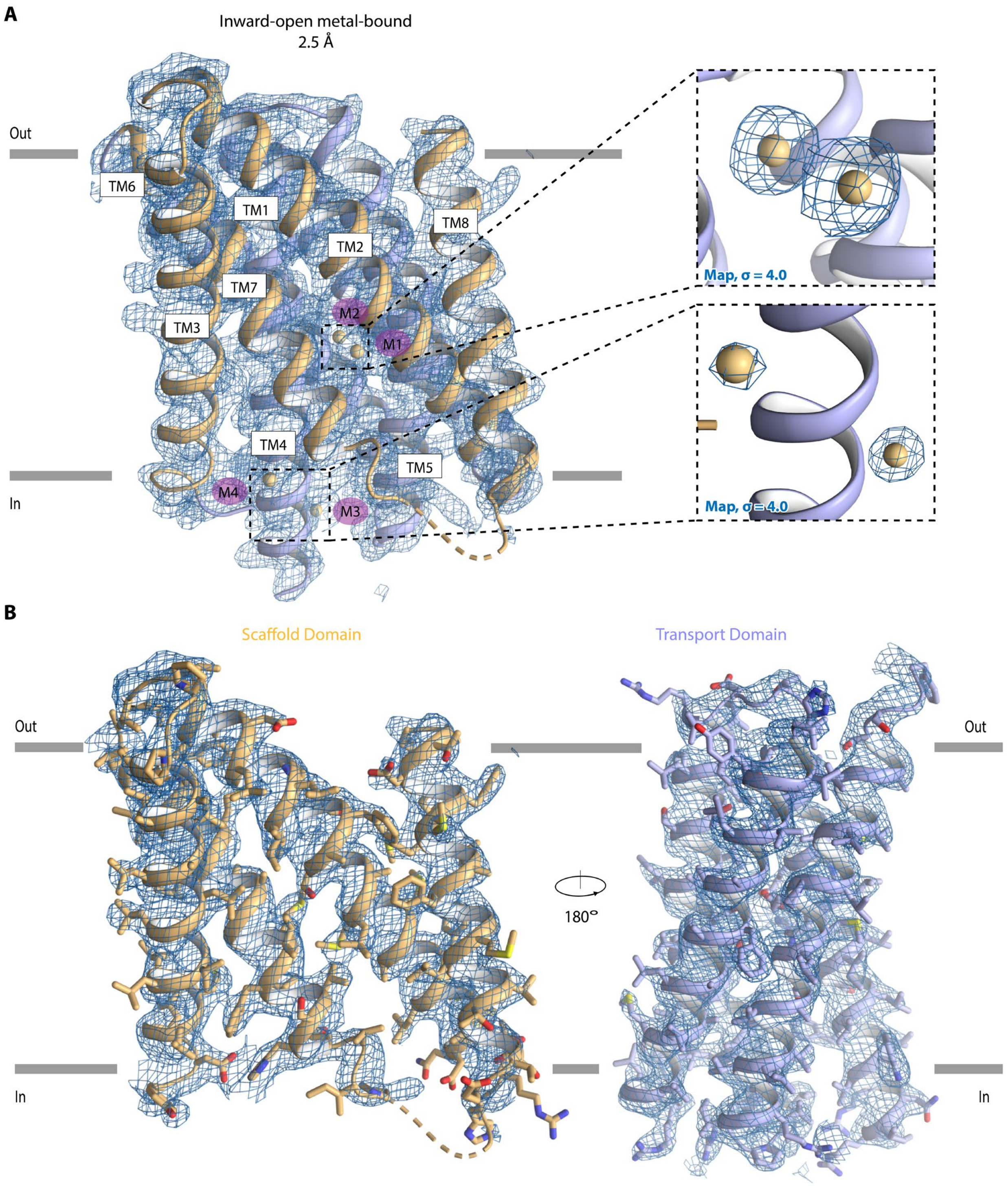

Fig. S2. **Electron density quality of the inward-open Cd^{2+}-bound structure determined here**. The final 2Fo-Fc electron density map contoured at σ=1.0 (blue mesh) if not stated otherwise. The overall resolution is indicated and the structures are colored as in Fig 1. **(A)** Overall structure of the inward-open metal-bound shown as cartoon with close views of the four metal-binding sites (σ=4.0). The view

is the same as in Fig 1. **(B)** Left panel: the scaffold domain shown in the same view as in panel A. Right panel: the transport domain as observed from the opposite side (180° rotation).

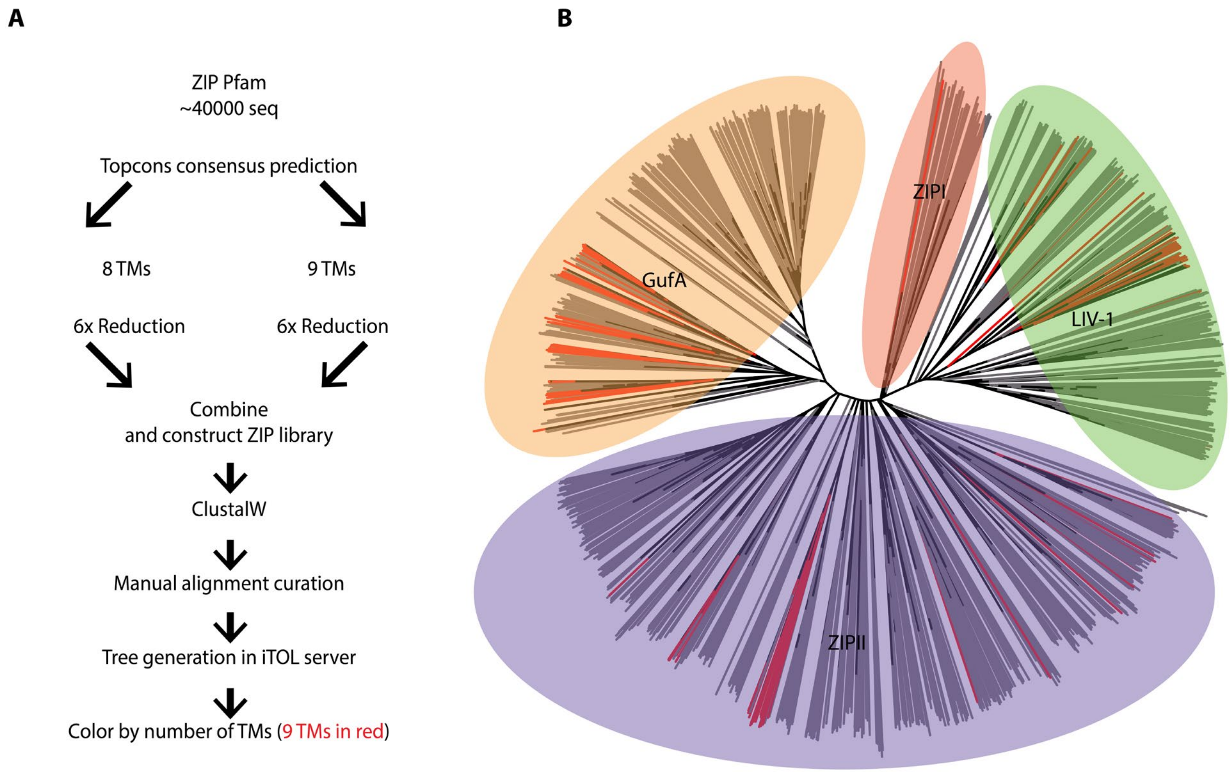

Fig. S3. Topology analysis of ZIPs. **(A)** Overview of topology analysis workflow. All proteins with the ZIP Pfam identifier were downloaded from the platform (http://pfam.xfam.org/) (*56*). These protein sequences were uploaded and analyzed with the consensus analysis tool TOPCONS (https://topcons.cbr.su.se/) (*57*). Each sequence was annotated with predicted topology, combined with sequences indicated in fig. S10 and aligned using the software ClustalW (http://www.clustal.org/clustal2/) (*58*). During the sequence alignment, a random reduction of the sequences was performed. The MSA was used to generate the phylogenetic tree with iTOL tool (https://itol.embl.de/) (*59*). **(B)** The ZIP sequences containing 9 TMs are label red in the tree. Interestingly, all four ZIP subfamilies, i.e, GufA, LIV-1, ZIPI and II (indicated by shaded ovals), have branches containing the 9-TM members.

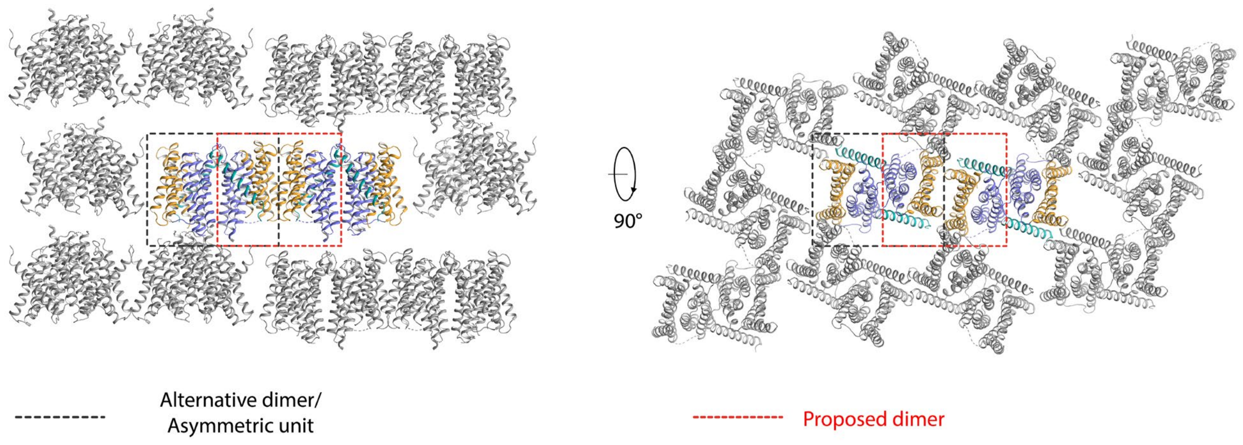

Fig. S4. The crystal packing of metal-free BbZIP includes two inside-in/inside-in dimers. The crystal packing is visualized in the plane of the membrane (left) and perpendicular to this view (right). The transmembrane helices are colored as in Fig. 1. The asymmetric unit displays a dimer with a dimer-interface between the two transports domains (black box). This alternative dimer formation is poorly explained by our EVcoupling analysis (Fig. 2) and is not in agreement with an elevator-type mechanism. However, inspection of the crystal packing reveals another (proposed) dimer, with a dimer-interface between the two scaffold domains (red box). The proposed dimer formation satisfies the inter-domain contacts in the EVcoupling analysis between TM3 and 8. Furthermore, this dimer interface also exhibits a hallmark of two-domain elevator-type transporters, interacting through a static scaffold domain. Surprisingly, the 'alternative dimer' is more stable (-18.6 kcal/mol) than the 'proposed dimer' (-4.9 kcal/mol), as indicated by PISA analysis (*60*).

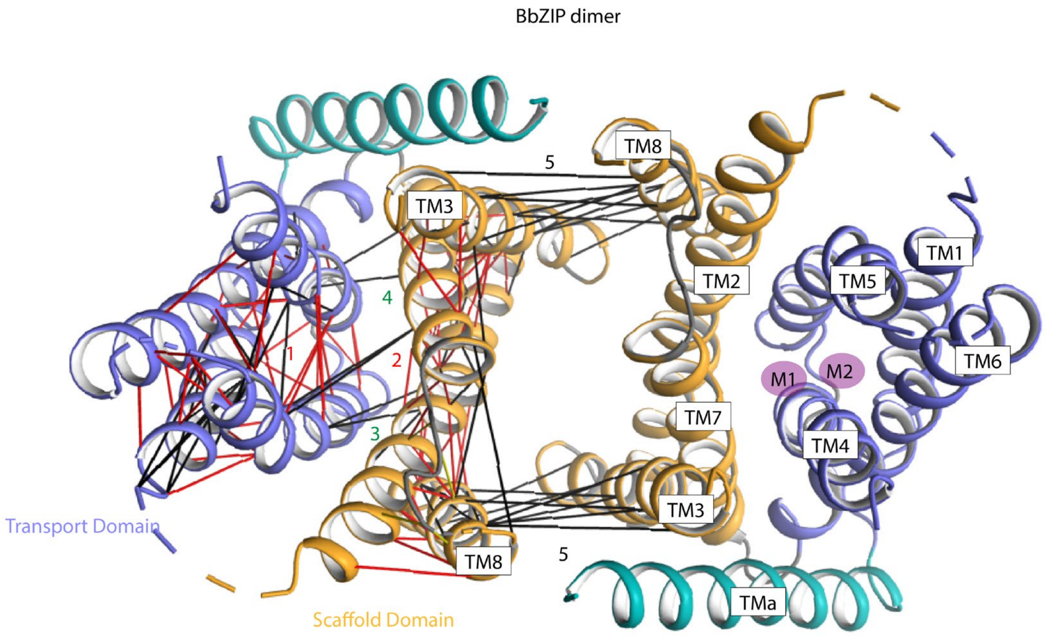

Fig. S5. Intra-helix-bundle interactions dominating in BbZIP. Analysis of evolutionary coupled amino acids was performed using the EVcouplings server (EVCS, https://evcouplings2.hms.harvard.edu/) (*19*) (see Fig. 2A). Evolutionary coupled pairs are connected by lines colored as in Fig 2A. Red lines shows evolutionary coupled pairs that are in contact in the 3D structure, dominating inside bundles (numbered 1 and 2, from Fig. 2A). Black lines (5) between TM3 and 8 displays interactions that agree with a homodimer supported by the scaffold domain. A few evolutionary coupled residues are also found in-between bundles (3 and 4). See also Fig. 2.

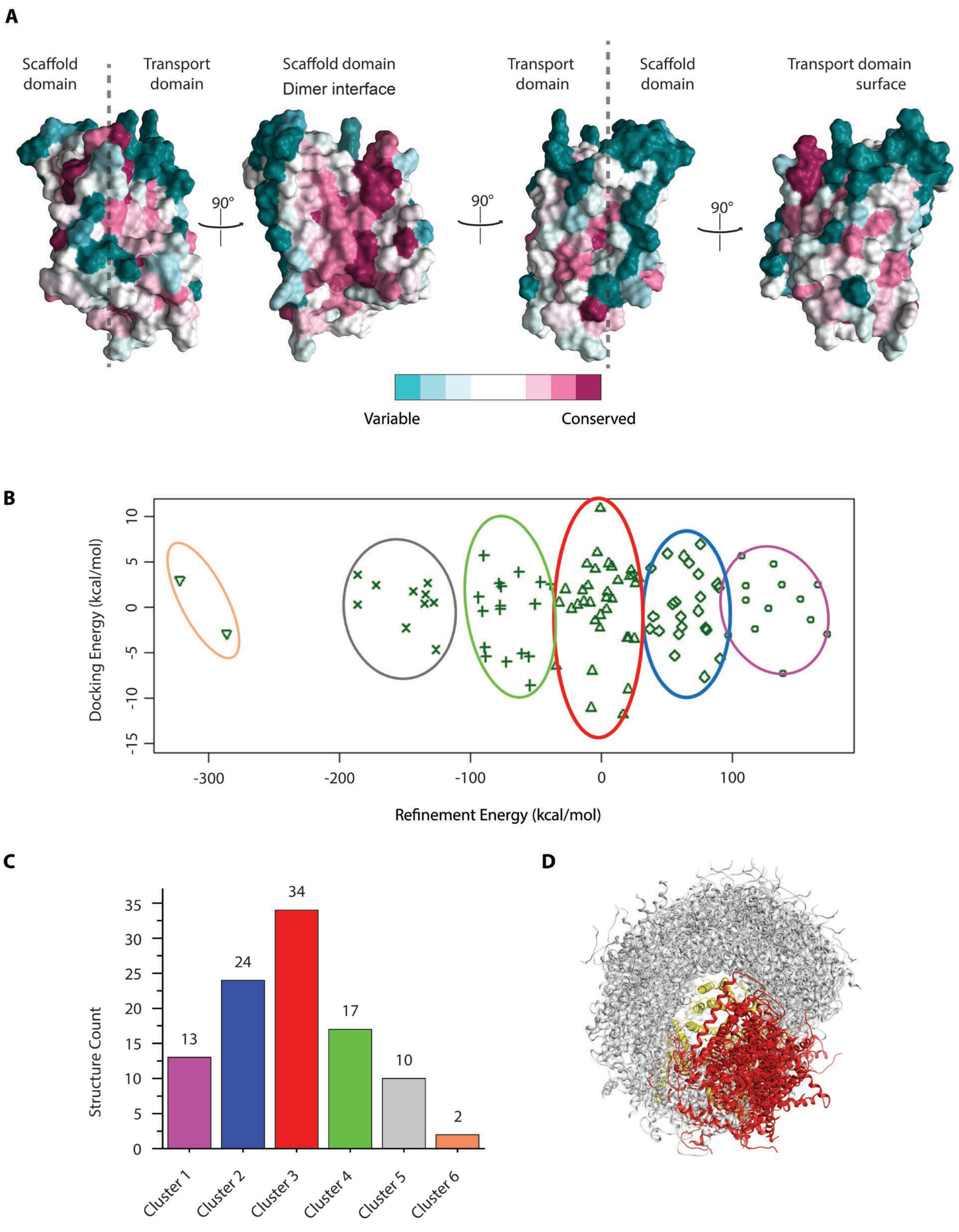

Fig. S6. Hypothetical dimer interfaces of BbZIP. (A) Four different surface views of the Conservation patterns in the BbZIP monomer. The membrane exposed part of the scaffold domain facing the

transport domain is much more conserved than the other regions. **(B)** Docking and refinement energies of the docked monomers. The identified clusters 1-6 are highlighted by the colored ovals. **(C)** The number of docking poses included in each cluster. **(D)** Docking poses of the highest populated cluster (#3) are depicted in red, with an average backbone root-mean-square deviation of 3.3 ± 0.8 Å to the proposed position and the other clusters displayed in white around a central monomeric BbZIP structure (shown in yellow).

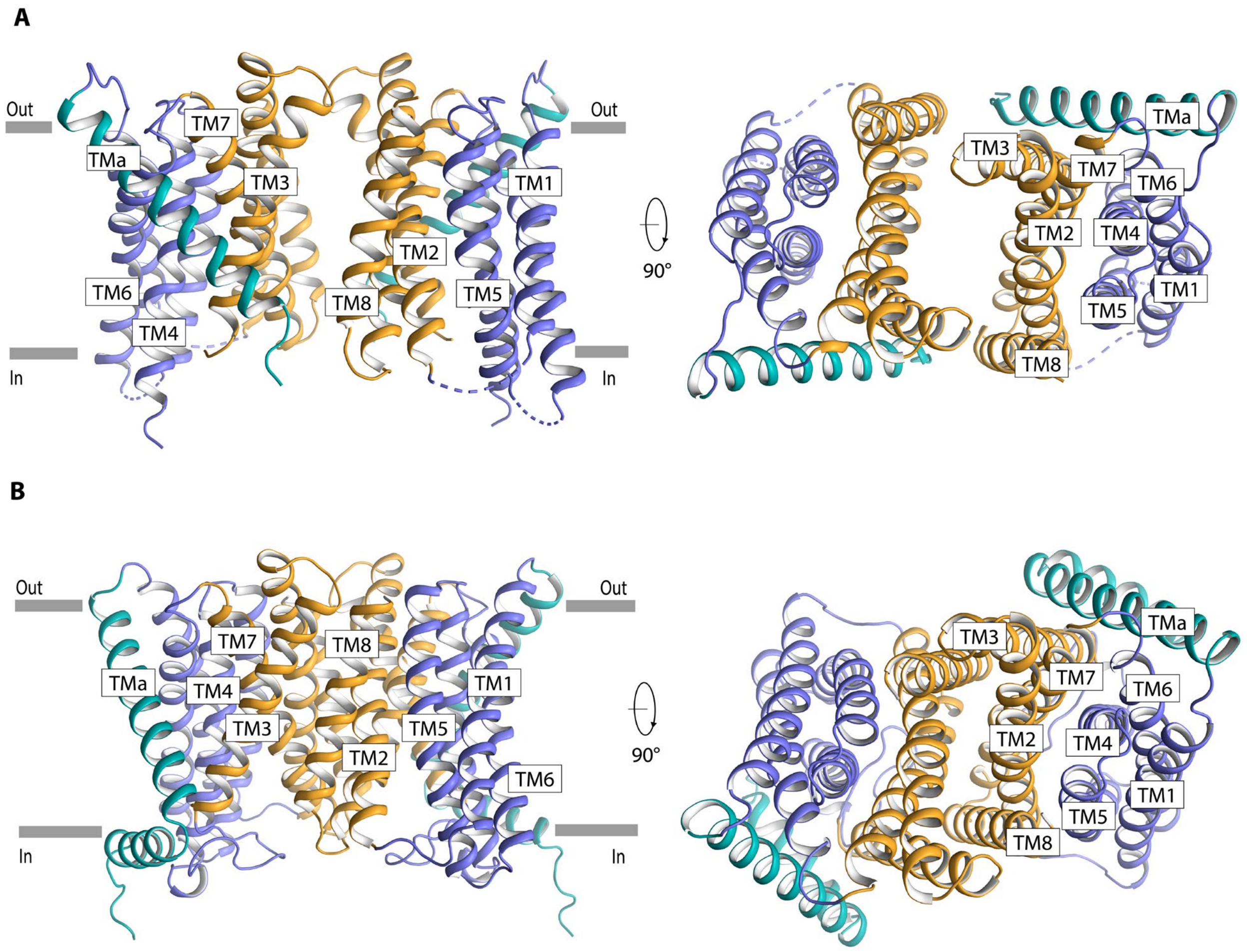

Fig. S7. Colabfold analysis supports the proposed dimer arrangement. (A) Proposed dimer formation of BbZIP as visible in the crystal packing of the inward-open metal-free structure. The antiparallel helix interactions between TM3 and 8 match the results from the EVcoupling analysis and agree with a homodimer formed through interactions between the two scaffold domains. **(B)** Structure predicted using the AlphaFold and RoseTTAFold-based tool, ColabFold. The structure shows a homodimer with interface interactions between the scaffold domain of each monomer as in the proposed dimer. However, compared to the dimer in panel (A), the interface in the ColabFold dimer is somewhat more tightly packed. This is also evident when analyzing the interface by PISA, as the solvation free energy gain upon interface formation is -35.4 kcal/mol. Noteworthy, due to the tighter packing, some of the evolutionary coupled residue pairs between TM7 and 8 are satisfied by the ColabFold structure. Views and coloring of TMs are the same as in fig. S4.

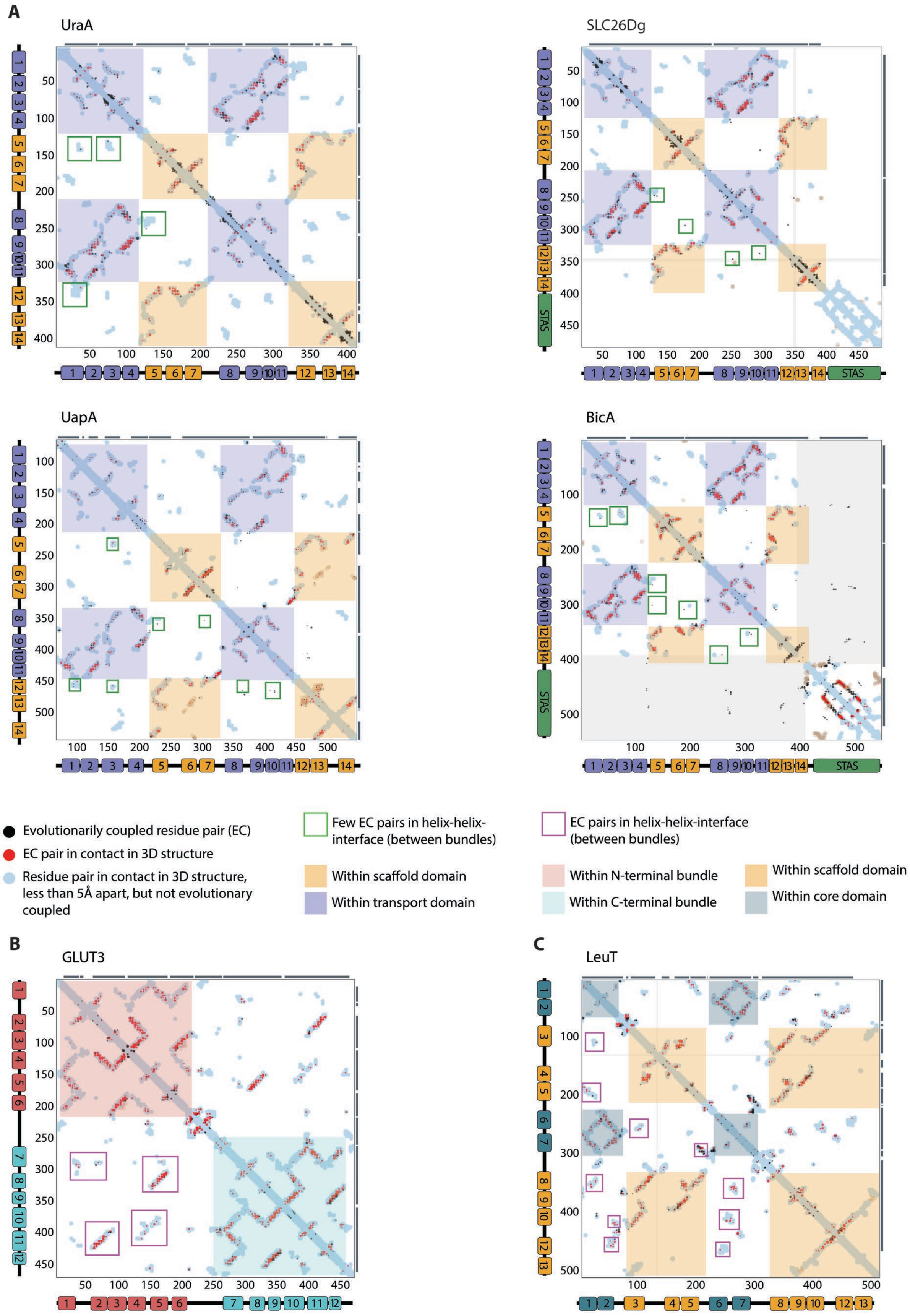
A
UraA
SLC26Dg
UapA
BicA
STAS
Evolutionarily coupled residue pair (EC)
EC pair in contact in 3D structure
Residue pair in contact in 3D structure, less than 5Å apart, but not evolutionary coupled
Few EC pairs in helix-helix-interface (between bundles)
EC pairs in helix-helix-interface (between bundles)
Within scaffold domain
Within transport domain
Within N-terminal bundle
Within C-terminal bundle
Within scaffold domain
Within core domain
B
GLUT3
C
LeuT

Fig S8. Evolutionary covariation analysis of transporters employing alternating access mechanisms. (A) Analysis of evolutionary coupled amino acids in elevator type transporters UraA, UapA, SLC26Dg and BicA respectively. The resulting contact map shows predicted evolutionary pairs (black dots) and structural contacts within 5 Å in the 3D structure (blue dots). Overlaps of EVCS-predicted constraints and monomer-available structural contacts are shown as red dots. Green boxes contain monomer-available helix interfaces with structural contacts between bundles. Importantly, inter-bundle helix interfaces are almost devoid of evolutionary coupled pairs, as also seen for BbZIP (Fig 2A). **(B)** and **(C)** Analysis of evolutionary coupled amino acids in the 'rocker switch' transporter GLUT3 and 'rocking bundle' transporter LeuT, respectively. Strikingly, the analysis displays many evolutionary coupled pairs in the inter-bundle helix interfaces of both GLUT3 and LeuT (purple boxes). Analysis of evolutionary coupled amino acids was performed using the EVcouplings server (EVCS, https://evcouplings2.hms.harvard.edu/) (*19*).

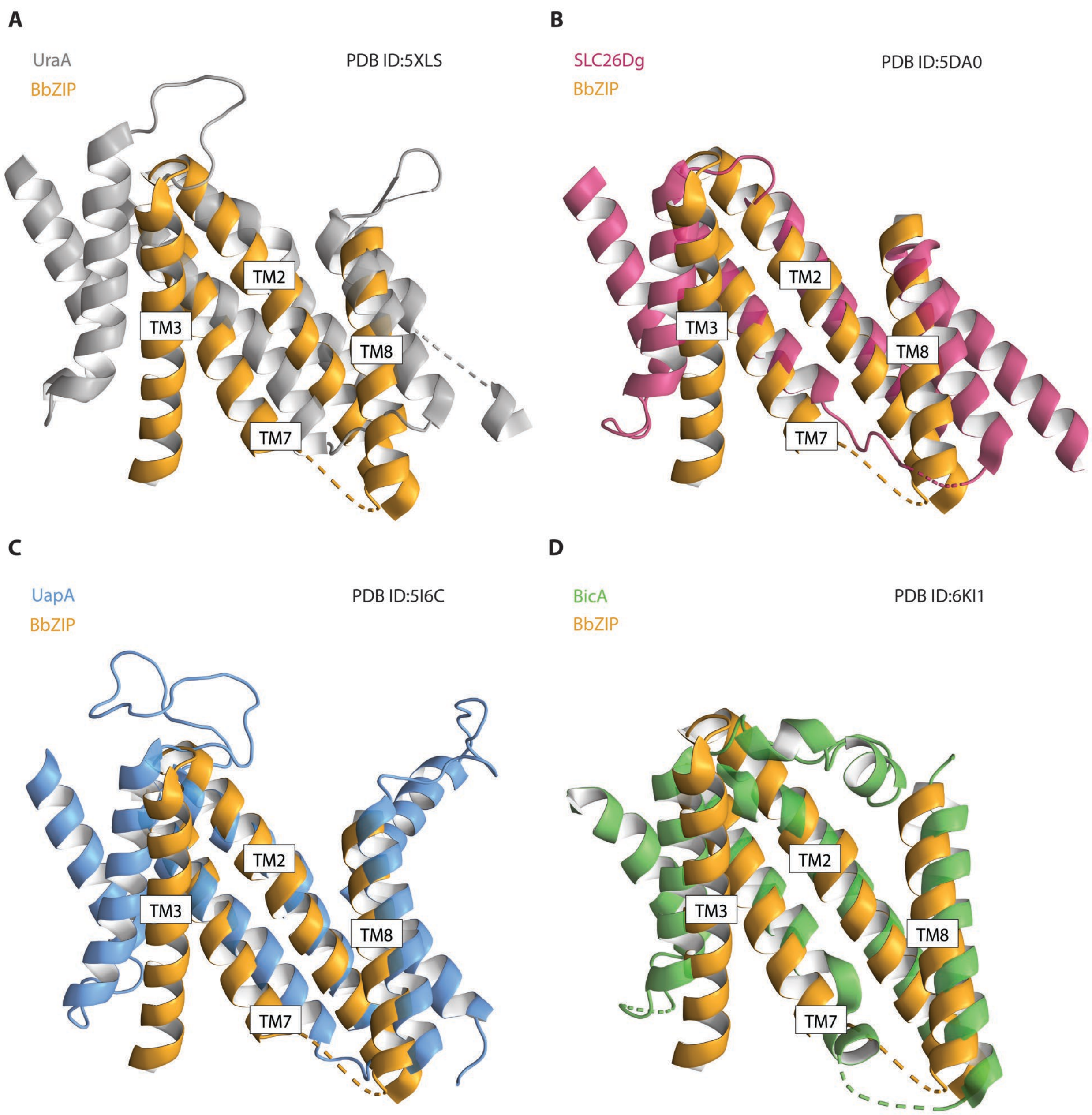

Fig. S9. Alignment of the scaffold domains of BbZIP and known elevator transporters. All transport domains have been removed for clarity. BbZIP is shown in orange in all panels. The elevator transporters analyzed in the evolutionary coupling analysis include: **(A)** UraA (PDB-ID: 5XLS) (*61*), **(B)** SLC26DG (PDB-ID: 5DA0) (*62*), **(C)** UapA (PDB-ID: 5I6C) (*63*) and **(D)** BicA (PDB-ID: 6KI1) (*29*). PDB-ID: protein data bank (https://www.rcsb.org/) (*64*) identification code.

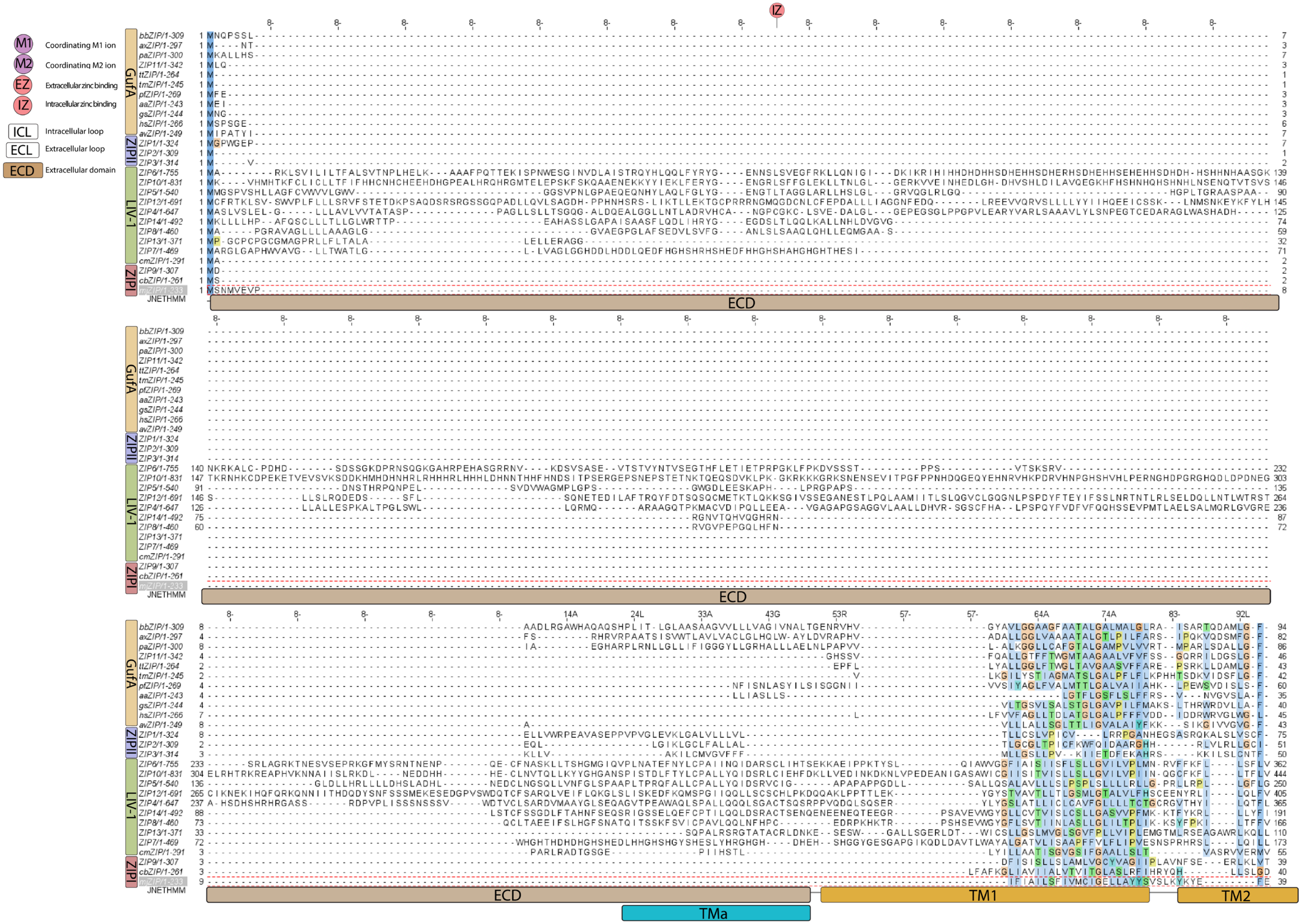

M1 Coordinating M1 ion
M2 Coordinating M2 ion
EZ Extracellular zinc binding
IZ Intracellular zinc binding
ICL Intracellular loop
ECL Extracellular loop
ECD Extracellular domain
GufA
ZIPII
LIV-1
ZIPI
JNETHMM
ECD
TMa
TM1
TM2

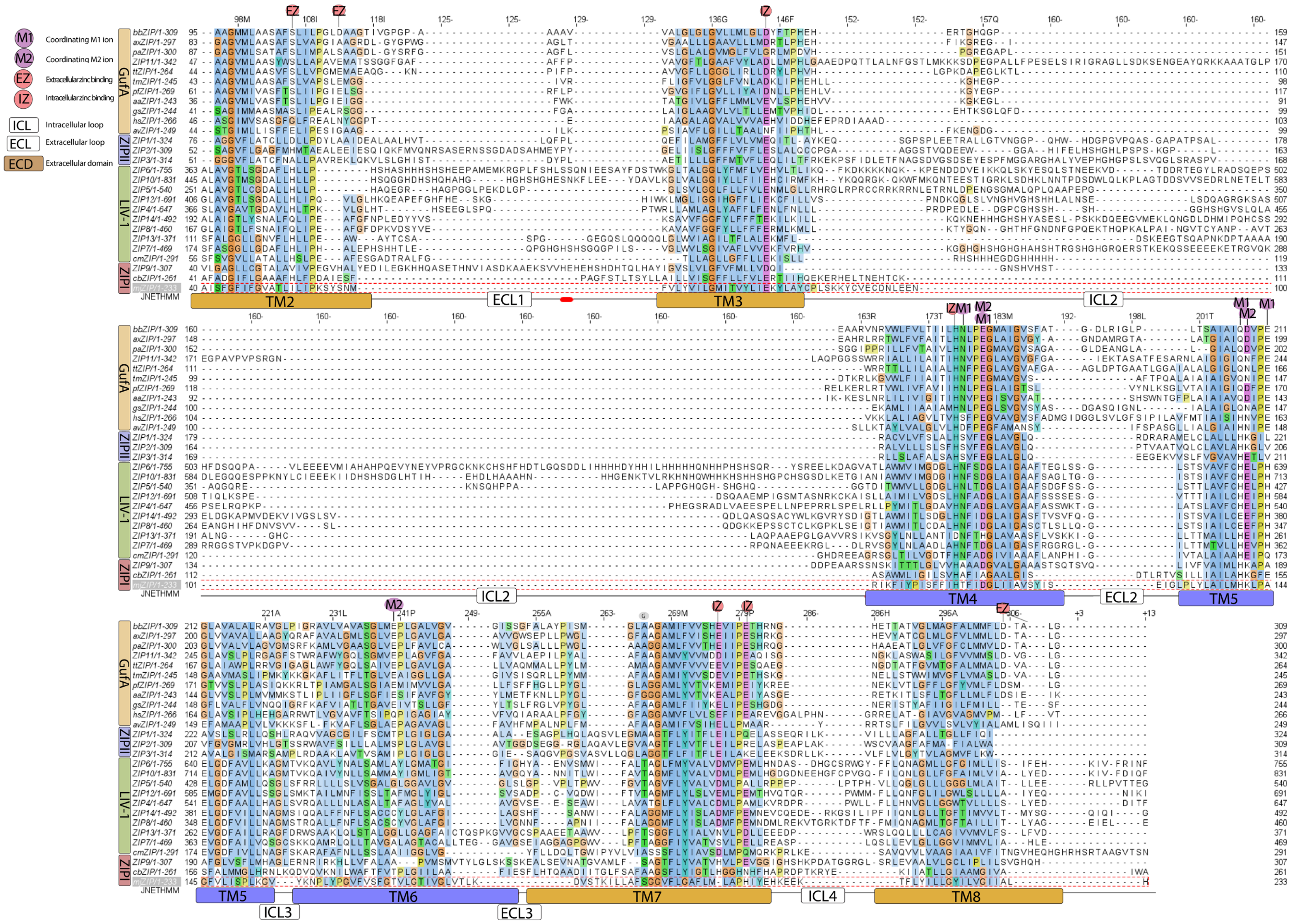
Coordinating M1 ion
Coordinating M2 ion
Extracellular zinc binding
Intracellular zinc binding
Intracellular loop
Extracellular loop
Extracellular domain
GufA
ZIPII
LIV-1
ZIPI
JNETHMM
TM2
TM3
TM4
TM5
TM6
TM7
TM8
ECL1
ECL2
ECL3
ICL2
ICL3
ICL4

Fig. S10. Alignment of selected ZIPs. Multiple sequence alignment of ZIPs of both bacterial and human origin, including all human members (ZIP1-14) and BbZIP. Residues coordinating the metal ion in the metal-binding sites (M1 and M2) are highlighted. ZIPs are divided into the four different subfamilies, i.e., GufA, LIV-1, ZIPI and II. Alignment was generated using the CLC Workbench and JalView.

A

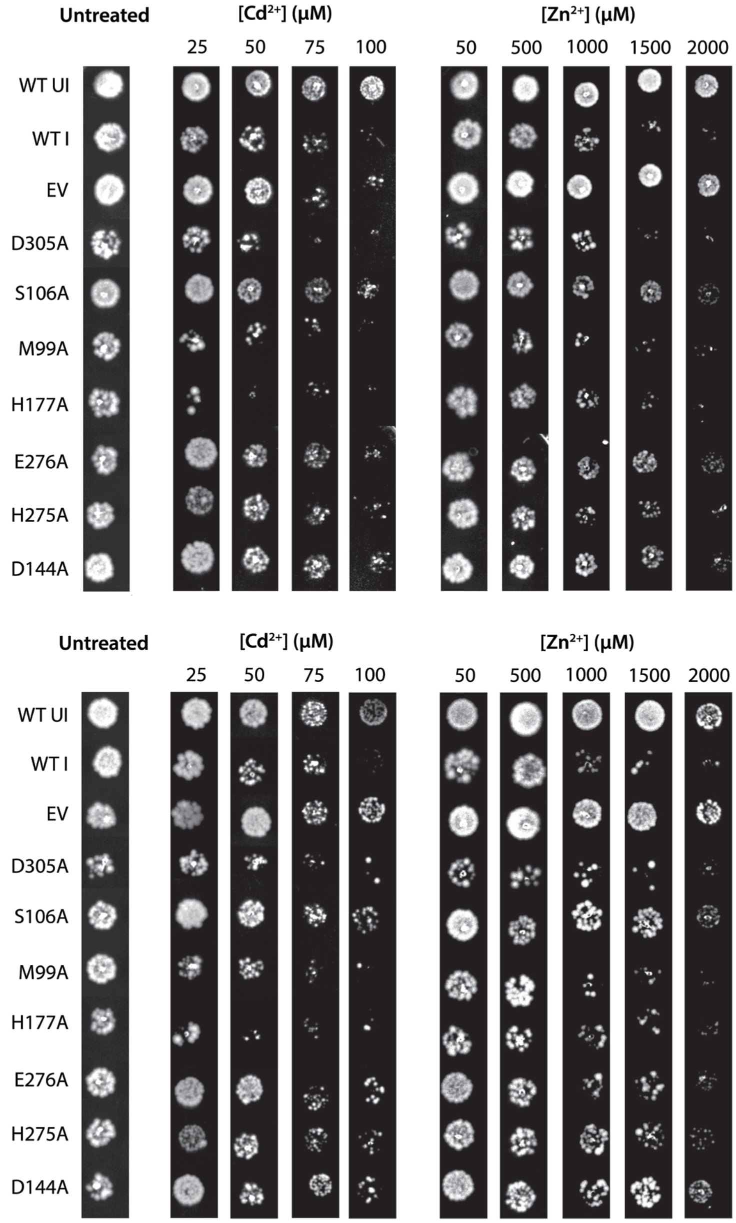
Untreated
[Cd2+] (µM)
25 50 75 100
[Zn2+] (µM)
50 500 1000 1500 2000
WT UI
WT I
EV
D305A
S106A
M99A
H177A
E276A
H275A
D144A
Untreated
[Cd2+] (µM)
25 50 75 100
[Zn2+] (µM)
50 500 1000 1500 2000
WT UI
WT I
EV
D305A
S106A
M99A
H177A
E276A
H275A
D144A

B

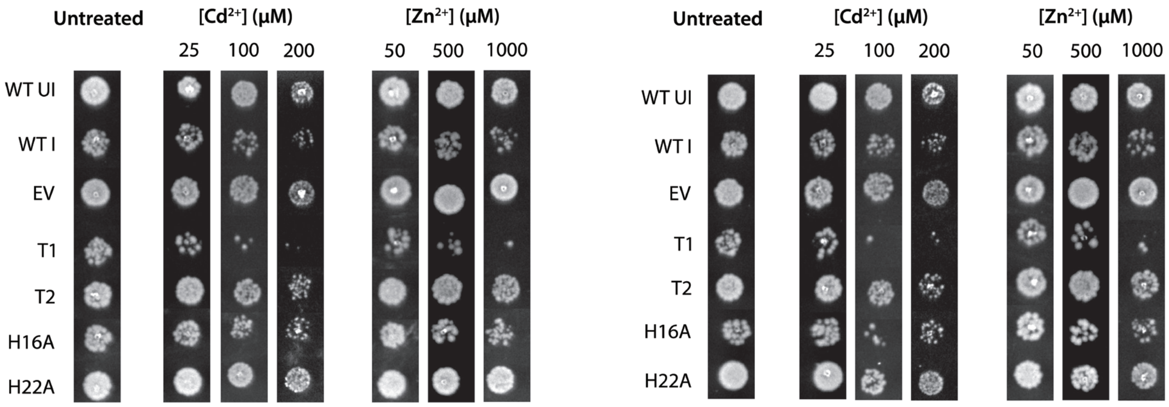
Untreated
[Cd2+] (µM)
25 100 200
[Zn2+] (µM)
50 500 1000
WT UI
WT I
EV
T1
T2
H16A
H22A
Untreated
[Cd2+] (µM)
25 100 200
[Zn2+] (µM)
50 500 1000
WT UI
WT I
EV
T1
T2
H16A
H22A

Fig. S11. Replicates of the cell-based toxicity assay. *Escherichia coli* cells (strain C43(DE3)) carrying either wild-type BbZIP (WT) single alanine mutations or truncations of the N-terminus were spotted on LB agar plates containing the indicated concentration of $ZnCl_2$ or $CdCl_2$ and incubated for 16 hours at 37 °C. Induced BbZIP (WT I) served as the positive control, whereas both uninduced BbZIP (WT UI) and empty vector (pET15b(+), EV) were included as negative controls. **(A)** Assayed alanine substitutions cover D305 and S106 of the extracellular vestibule, E276, H275 and D144 of the intracellular bowl, and M99 and H177 in the bundle-bundle-interface. See Fig. 3A and 3B for location of the mutated residues in BbZIP. **(B)** Assayed variants include two N-terminal truncations, i.e., T1 (Δ3-20) and T2 (Δ3-52), and two single mutants, i.e., H16A and H22A. The third replicate is shown in figures 5 and 6. Immunoblots showing the expression level of WT and mutants are presented in Fig. 6B and fig. S14.

A

Family	Protein examples	Transport mode	Cargo	TM4			TM5			TM6
				1	2	3	1	2	3	1
GufA	BbZIP	Passive transport	Zn^{2+}	His177	Asn178	Glu181	Gln207	Asp208	Glu211	Glu240
	hZIP11	Passive transport	Zn^{2+}	His	Asn	Glu	Gln	Asn	Glu	Glu
ZIPII	hZIP1/hZIP2/hZIP3	Passive transport	Zn^{2+}	His	Ser	Glu	His	Lys	Leu/Val	Ser/Thr
LIV-1	hZIP4/hZIP6/hZIP10	Co-transport	Zn^{2+}/H^{+}	His	Asn	Asp	His	Glu	His	Ala
	hZIP8/hZIP14	Co-transport	Zn^{2+}/HCO_3^-	His	Asn	Asp	His	Glu	His	Cys
ZIPI	hZIP9	?	Zn^{2+}(/?)	His	Ala	Asp	His	Lys	Ala	Ala

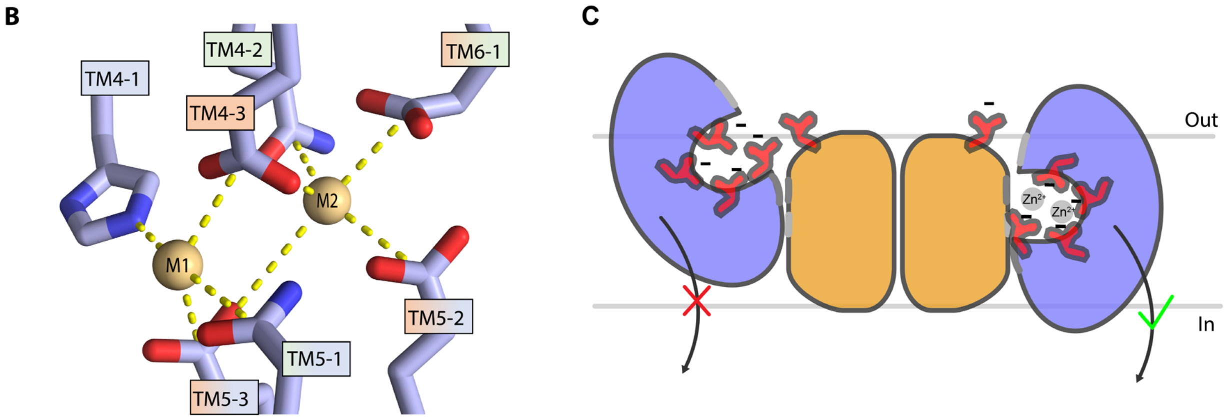

Fig. S12. Ion-binding region and charge compensation model. (A) Table of important zinc-coordinating residues in the ion-binding region of selected ZIPs belonging to the different subfamilies. Indicated residues are present in TM4, 5 and 6, and are color-coded based on chemical properties. Positively charged residues are shown in blue, polar in green, negatively charged in red and hydrophobic in yellow, respectively. **(B)** Structural presentation of the coordination network of ion-binding site residues. Shown residues are colored and labelled as in panel A. ZIP1, 2, 3 and 9 lack the M2 binding site, as it is occupied by a charged lysine residue in position TM5-2. **(C)** Charge compensation model. There is a significant energetic penalty of transporting charges across the membrane. In the absence of zinc, the ion-binding region of ZIPs is heavily negatively charged (left monomer), preventing movements of the transport domain. Upon zinc binding (right monomer), the binding site becomes electroneutral. This may permit stochastic movements of the transport domain across the membrane, minimizing the large electrostatic penalty required to transfer charges through the membrane.

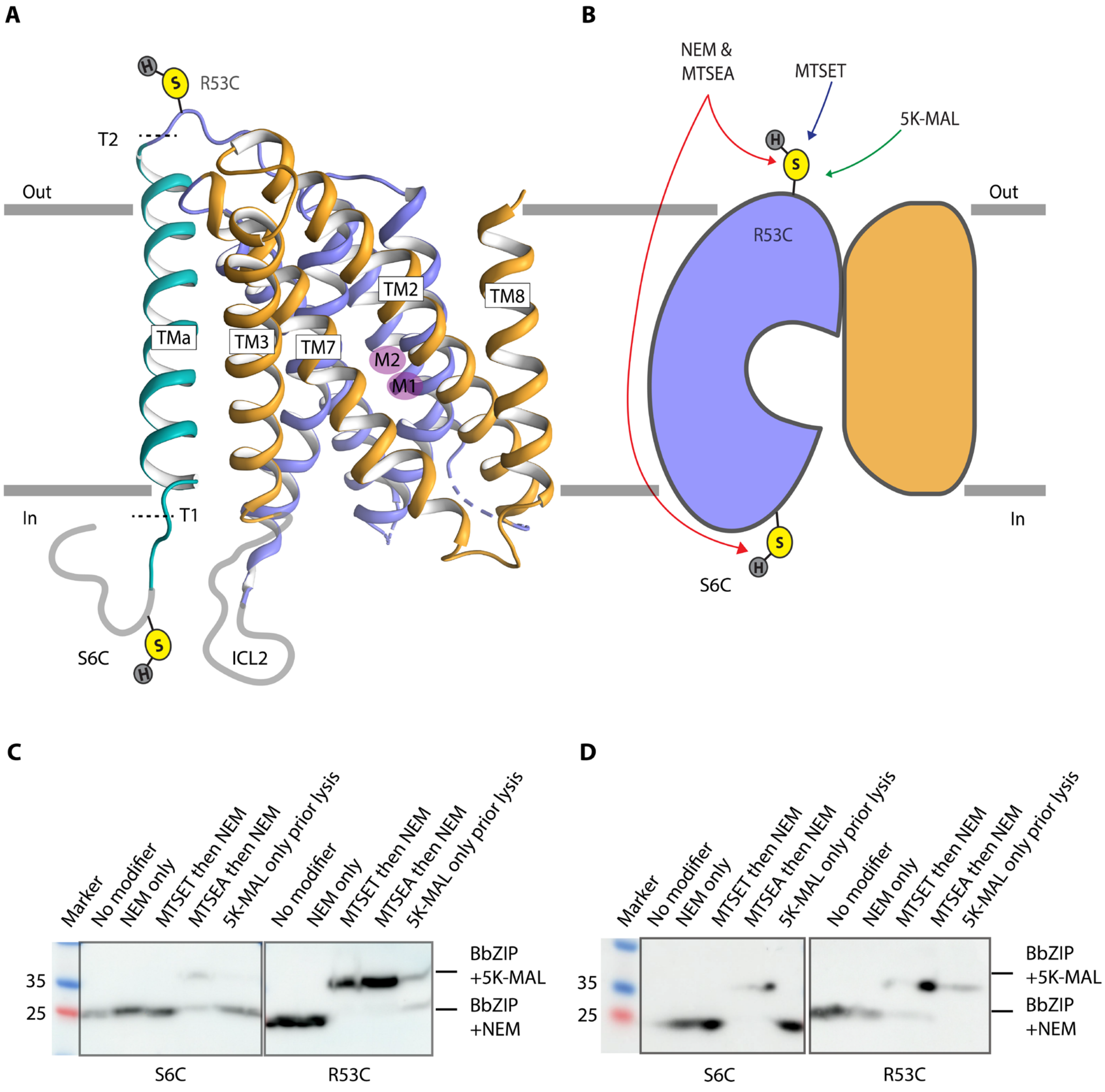

Fig. S13. The cysteine accessibility assay and additional replicate. (A) BbZIP topology and placement of single cysteine residues introduced to assess the location of TMa. **(B)** Overview of the cysteine accessibility assay. To assess the presence of TMa *in vivo* and map its topology, two separate single mutants introducing a cysteine residue on each side of TMa were generated. S6C serves as an inside reporter, whereas R53C is an outside probe. To determine whether the cysteines are accessible from the periplasm or the cytosol, MTSET that is impermeable to the membrane and MTSEA that is permeable to the membrane was utilized. MTSET or MTSEA was added prior to treatment with N-ethylmaleimide (NEM). NEM blocked cysteines accessible not bound by MTSET or MTSEA. Reversible disulfide bonds of MTSET or MTSEA were removed by addition of DTT in the denaturation step.

Conversely, NEM reacts with exposed cysteine residues in an irreversible manner (if not protected by MTSET or MTSEA). Hence, only cysteines located on the extracellular side will react with MTSET, while all accessible cysteines will interact with MTSEA. Following exposure to DTT, accessible cysteines will react with 5K-MAL, resulting in increasing the total molecular weight and decreasing electrophoretic mobility, detectable as up-shift of the protein band in the immunoblot. **(C)** Immunoblot analysis of S6C and R53C BbZIP mutants in the cysteine accessibility assay. Samples in lanes 1 and 6 were untreated, in lanes 2 and 7 were treated with NEM, and subsequently with 5K-MAL. Samples in lanes 3 and 8 were exposed to MTSET, followed by NEM and 5K-MAL treatments while samples in lanes 4 and 9 were exposed to MTSEA, followed by NEM and 5K-MAL treatments. Samples in lanes 5 and 10 were treated only with 5K-MAL prior to cell lysis. Results show an up-shift of the protein band for the R53C mutant when treated with both MTSET and MTSEA, while S6C only experience an up-shift when treated with MTSEA. Thus, S6C is located intracellularly *in vivo*. **(D)** Replicate of the cysteine accessibility assay. Immunoblot analysis of S6C and R53C BbZIP mutants in the cysteine accessibility assay (Fig. 8). Samples in lanes 1 and 6 were untreated, in lanes 2 and 7 were treated with NEM, and subsequently with 5K-MAL. Samples in lanes 3 and 8 were exposed to MTSET, followed by NEM and 5K-MAL treatments while samples in lanes 4 and 9 were exposed to MTSEA, followed by NEM and 5K-MAL treatments. Samples in lanes 5 and 10 were treated only with 5K-MAL prior to cell lysis. Results show an up-shift of the protein band for the R53C mutant when treated with both MTSET and MTSEA, while S6C only experience an up-shift when treated with MTSEA. Thus, S6C is located intracellularly *in vivo*.

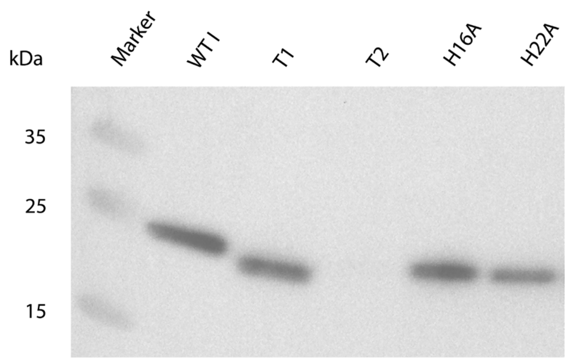

Fig. S14. Immunoblot analysis of TMa mutants. The corresponding crude lysates originating from the *Escherichia coli* cells assayed in the *in vivo* metal toxicity assay (Fig. 7B) were resolved on SDS-PAGE, blotted, and visualized using an anti-His$_6$-tag antibody conjugated with horseradish peroxidase. The tested alanine forms and the truncated construct T1 (Δ3-20) were produced at comparable levels as WT BbZIP, while the T2 (Δ3-52) variant was not produced. Representative results from a single experiment repeated three times are shown.

Table S1. Data collection and refinement statistics. Statistics for the highest-resolution shell are shown in parentheses.

	BbZIP<br>Cadmium-bound inward-open<br>PDB-ID: 7Z6M	BbZIP<br>Metal-free inward-open<br>PDB-ID: 7Z6N
**Wavelength**	1.0	1.0
**Resolution range**	45.51 - 2.51 (2.6 - 2.51)	41.17- 2.57 (2.66 – 2.57)
**Space group**	C 1 2 1	C 2 2 21
**Unit cell**	96.211 61.656 55.262 90 108.898 90	111.493 122.117 101.733 90 90 90
**Total reflections**	79759 (7190)	344302 (33115)
**Unique reflections**	10448 (1011)	22269 (663)
**Multiplicity**	7.6 (7.1)	15.5 (15.1)
**Completeness (%)**	98.62 (96.65)	89.77 (29.81)
**Mean I/sigma(I)**	8.56 (0.73)	5.02 (0.04)
**Wilson B-factor**	79.76	101.32
**R-merge**	0.1628 (2.229)	0.4669 (24.74)
**R-meas**	0.1751 (2.403)	0.4831 (25.6)
**R-pim**	0.06329 (0.8814)	01222 (6.51)
**CC1/2**	0.998 (0.41)	0.999 (0.0526)
**CC***	0.999 (0.762)	1 (0.316)
**Reflections used in refinement**	10429 (1010)	14399 (210)
**Reflections used for R-free**	1039 (100)	745 (5)
**R-work**	0.2295 (0.3811)	0.2872 (0.3567)
**R-free**	0.2783 (0.3824)	0.3355 (0.5900)
**CC(work)**	0.952 (0.662)	0.898 (0.451)
**CC(free)**	0.984 (0.346)	0.917 (0.403)
**Number of non-hydrogen atoms**	1662	3785
**macromolecules**	1658	35745
**ligands**	4	30
**solvent**		10
**Protein residues**	238	536
**RMS(bonds)**	0.008	0.002
**RMS(angles)**	1.10	0.73
**Ramachandran favored (%)**	96.12	97.14
**Ramachandran allowed (%)**	3.88	2.67
**Ramachandran outliers (%)**	0.00	0.19
**Rotamer outliers (%)**	0.62	0.00
**Clashscore**	12.32	1.56
**Average B-factor**	90.69	67.16
**macromolecules**	90.62	66.94
**ligands**	117.68	96.76
**solvent**		60.68

Table S2. Sequences of cloning primers used to engineer mutants of BbZIP.

**N-terminal truncations**	
T1 (Δ3-20)_fw	5'-GCGCGGCAGCCATATGAATAGCCATCCGC-3'
T1 (Δ3-20)_rev	5'-GCGGATGGCTATTCATATGGCTGCCGCGC-3'
T1 (Δ3-20)_rev	5'-GCGGATGGCTATTCATATGGCTGCCGCGC-3'
T2 (Δ3-52)_fw	5'-CGCGGCAGCCATATGAACCGTGTGCACGTG-3'
T2 (Δ3-52)_rev	5'-CACGTGCACACGGTTCATATGGCTGCCGCG-3'
**N-terminus**	
H16A_fw	5'-GCGTGGTGCGTGGGCTGCGCAAGCGCAAAG-3'
H16A_rev	5'-CTTTGCGCTTGCGCAGCCCACGCACCACGC-3'
H22A_fw	5'-GCGCAAGCGCAAAGCGCTCCGCTGATTACCCT-3'
H22A_rev	5'-AGGGTAATCAGCGGAGCGCTTTGCGCTTGCGC-3'
**Metal transport pathway**	
S106A_fw	5'-TGGCGGCGAGCGCGTTTGCCCTGATTCTGC-3'
S106A_rev	5'-GCAGAATCAGGGCAAACGCGCTCGCCGCCAGC-3'
D305A_fw	5'-GATGTTCCTGGCCACCGCGCTGGGCTAACTCG-3'
D305A_rev	5'-CGAGTTAGCCCAGCGCGGTGGCCAGGAACATC-3'
D144A_fw	5'-CTGATGCTGGGTCTGGCCTATTTTACCCCGCAC-3'
D144A_rev	5'-GTGCGGGGTAAAATAGGCCAGACCCAGCATCAG-3'
H275A_fw	5'-GGTTTCCGGAATCACTTCGGCGCTAACCACAAAGATCATC-3'
H275A_rev	5'-GATGATCTTTGTGGTTAGCGCCGAAGTGATTCCGGAAACC-3'
E276A_fw	5'-CTTTGTGGTTAGCCACGCAGTGATTCCGGAAACCC-3'
E276A_rev	5'-GGGTTTCCGGAATCACTGCGTGGCTAACCACAAAG-3'
**Metal-binding sites**	
M99A_fw	5'-GCTCGCCGCCAGCGCCATACCCGCCGCA-3'
M99A_rev	5'-TGCGGCGGGTATGGCGCTGGCGGCGAGC-3'
H177A_fw	5'-TCTGACCATCATTCTGGCCAACCTGCCGGAAGGC-3'
H177A_rev	5'-GCCTTCCGGCAGGTTGGCCAGAATGATGGTCAGA-3'

REFERENCES AND NOTES

1. T. Hara, T.-A. Takeda, T. Takagishi, K. Fukue, T. Kambe, T. Fukada, Physiological roles of zinc transporters: Molecular and genetic importance in zinc homeostasis. *J. Physiol. Sci.* **67**, 283–301 (2017).

2. C. Andreini, L. Banci, I. Bertini, A. Rosato, Counting the zinc-proteins encoded in the human genome. *J. Proteome Res.* **5**, 196–201 (2006).

3. B. H. Bin, J. Seo, S. T. Kim, Function, structure, and transport aspects of ZIP and ZnT Zinc transporters in immune cells. *J. Immunol. Res.*. **2018**, 9365747 (2018).

4. S. Küry, B. Dréno, S. Bézieau, S. Giraudet, M. Kharfi, R. Kamoun, J. P. Moisan, Identification of SLC39A4, a gene involved in acrodermatitis enteropathica. *Nat. Genet.* **31**, 239–240 (2002).

5. T. Fukada, N. Civic, T. Furuichi, S. Shimoda, K. Mishima, H. Higashiyama, Y. Idaira, Y. Asada, H. Kitamura, S. Yamasaki, S. Hojyo, M. Nakayama, O. Ohara, H. Koseki, H. G. dos Santos, L. Bonafe, R. Ha-Vinh, A. Zankl, S. Unger, M. E. Kraenzlin, J. S. Beckmann, I. Saito, C. Rivolta, S. Ikegawa, A. Superti-Furga, T. Hirano, The zinc transporter SLC39A13/ZIP13 is required for connective tissue development; its involvement in BMP/TGF-β signaling pathways. *PLOS ONE* **3**, e3642 (2008).

6. L. A. Gaither, D. J. Eide, Functional expression of the human hZIP2 zinc transporter. *J. Biol. Chem.* **275**, 5560–5564 (2000).

7. L. He, K. Girijashanker, T. P. Dalton, J. Reed, H. Li, M. Soleimani, D. W. Nebert, ZIP8, member of the solute-carrier-39 (SLC39) metal-transporter family: Characterization of transporter properties. *Mol. Pharmacol.* **70**, 171–180 (2006).

8. K. Girijashanker, L. He, M. Soleimani, J. M. Reed, H. Li, Z. Liu, B. Wang, T. P. Dalton, D. W. Nebert, Slc39a14 gene encodes ZIP14, a metal/bicarbonate symporter: Similarities to the ZIP8 transporter. *Mol. Pharmacol.* **73**, 1413–1423 (2008).

9. L. A. Gaither, D. J. Eide, The human ZIP1 transporter mediates zinc uptake in human K562 erythroleukemia cells. *J. Biol. Chem.* **276**, 22258–22264 (2001).

10. Z. Liu, H. Li, M. Soleimani, K. Girijashanker, J. M. Reed, L. He, T. P. Dalton, D. W. Nebert, Cd^{2+} versus Zn^{2+} uptake by the ZIP8 HCO_3^--dependent symporter: Kinetics, electrogenicity and trafficking. *Biochem. Biophys. Res. Commun.* **365**, 814–820 (2008).

11. E. Hoch, M. Levy, M. Hershfinkel, I. Sekler, Elucidating the H^+ coupled Zn^{2+} transport mechanism of ZIP4; implications in acrodermatitis enteropathica. *Int. J. Mol. Sci.* **21**, 734 (2020).

12. G. Gyimesi, G. Albano, D. G. Fuster, M. A. Hediger, J. Pujol-Giménez, Unraveling the structural elements of pH sensitivity and substrate binding in the human zinc transporter SLC39A2 (ZIP2). *J. Biol. Chem.* **294**, 8046–8063 (2019).

13. W. Lin, J. Chai, J. Love, D. Fu, Selective electrodiffusion of zinc ions in a Zrt-, Irt-like protein, ZIPB. *J. Biol. Chem.* **285**, 39013–39020 (2010).

14. B. H. Bin, T. Fukada, T. Hosaka, S. Yamasaki, W. Ohashi, S. Hojyo, T. Miyai, K. Nishida, S. Yokoyama, T. Hirano, Biochemical characterization of human ZIP13 protein: A homo-dimerized zinc transporter involved in the spondylocheiro dysplastic Ehlers-Danlos syndrome. *J. Biol. Chem.* **286**, 40255–40265 (2011).

15. T. P. Ajeesh Krishna, T. Maharajan, G. Victor Roch, S. Ignacimuthu, S. Antony Ceasar, Structure, function, regulation and phylogenetic relationship of ZIP family transporters of plants. *Front. Plant. Sci.* **11**, 662 (2020).

16. T. Zhang, J. Liu, M. Fellner, C. Zhang, D. Sui, J. Hu, Crystal structures of a ZIP zinc transporter reveal a binuclear metal center in the transport pathway. *Sci. Adv.* **3**, e1700344 (2017).

17. T. Zhang, D. Sui, C. Zhang, L. Cole, J. Hu, Asymmetric functions of a binuclear metal center within the transport pathway of a human zinc transporter ZIP4. *FASEB J.* **34**, 237–247 (2020).

18. T. Zhang, D. Sui, J. Hu, Structural insights of ZIP4 extracellular domain critical for optimal zinc transport. *Nat. Commun.* **7**, 11979 (2016).

19. T. A. Hopf, A. G. Green, B. Schubert, S. Mersmann, C. P. I. Schärfe, J. B. Ingraham, A. Toth-Petroczy, K. Brock, A. J. Riesselman, P. Palmedo, C. Kang, R. Sheridan, E. J. Draizen, C. Dallago, C.

Sander, D. S. Marks, The EVcouplings Python framework for coevolutionary sequence analysis. *Bioinformatics* **35**, 1582–1584 (2019).

20. M. Mirdita, S. Ovchinnikov, M. Steinegger, ColabFold—Making protein folding accessible to all. bioRxiv 10.1101/2021.08.15.456425 [**Preprint**]. 2021.

21. R. Tibshirani, T. Hastie, B. Narasimhan, G. Chu, Diagnosis of multiple cancer types by shrunken centroids of gene expression. *Proc. Natl. Acad. Sci. U.S.A.* **99**, 6567–6572 (2002).

22. S. Antala, S. Ovchinnikov, H. Kamisetty, D. Baker, R. E. Dempski, Computation and functional studies provide a model for the structure of the zinc transporter hZIP4. *J. Biol. Chem.* **290**, 17796–17805 (2015).

23. F. M. Richards, C. E. Kundrot, Identification of structural motifs from protein coordinate data: Secondary structure and first-level supersecondary structure. *Proteins* **3**, 71–84 (1988).

24. A. A. Garaeva, D. J. Slotboom, Elevator-type mechanisms of membrane transport. *Biochem. Soc. Trans.* **48**, 1227–1241 (2020).

25. D. Drew, O. Boudker, Shared molecular mechanisms of membrane transporters. *Annu. Rev. Biochem.* **85**, 543–572 (2016).

26. T. A. Hopf, L. J. Colwell, R. Sheridan, B. Rost, C. Sander, D. S. Marks, Three-dimensional structures of membrane proteins from genomic sequencing. *Cell* **149**, 1607–1621 (2012).

27. B. Byrne, It takes two to transport via an elevator. *Cell Res.* **27**, 965–966 (2017).

28. C. Lee, H. J. Kang, C. von Ballmoos, S. Newstead, P. Uzdavinys, D. L. Dotson, S. Iwata, O. Beckstein, A. D. Cameron, D. Drew, A two-domain elevator mechanism for sodium/proton antiport. *Nature* **501**, 573–577 (2013).

29. C. Wang, B. Sun, X. Zhang, X. Huang, M. Zhang, H. Guo, X. Chen, F. Huang, T. Chen, H. Mi, F. Yu, L. N. Liu, P. Zhang, Structural mechanism of the active bicarbonate transporter from cyanobacteria. *Nat. Plants* **5**, 1184–1193 (2019).

30. B. H. Thurtle-Schmidt, R. M. Stroud, Structure of Bor1 supports an elevator transport mechanism for SLC4 anion exchangers. *Proc. Natl. Acad. Sci. U.S.A.* **113**, 10542–10546 (2016).

31. M. Hirschi, Z. L. Johnson, S.-Y. Lee, Visualizing multistep elevator-like transitions of a nucleoside transporter. *Nature* **545**, 66–70 (2017).

32. M. Duffield, A. Patel, O. V. Mortensen, D. Schnur, A. D. Gonzalez-Suarez, D. Torres-Salazar, A. C. K. Fontana, Transport rate of EAAT2 is regulated by amino acid located at the interface between the scaffolding and substrate transport domains. *Neurochem. Int.* **139**, 104792 (2020).

33. D. B. Sauer, N. Trebesch, J. J. Marden, N. Cocco, J. Song, A. Koide, S. Koide, E. Tajkhorshid, D.-N. Wang, Structural basis for the reaction cycle of DASS dicarboxylate transporters. *eLife* **9**, e61350 (2020).

34. J. S. Lolkema, D. J. Slotboom, Structure and elevator mechanism of the Na^+-citrate transporter CitS. *Curr. Opin. Struct. Biol.* **45**, 1–9 (2017).

35. C. Grewer, Z. Zhang, J. Mwaura, T. Albers, A. Schwartz, A. Gameiro, Charge compensation mechanism of a Na^+-coupled, secondary active glutamate transporter. *J. Biol. Chem.* **287**, 26921–26931 (2012).

36. P. Uzdavinys, M. Coinçon, E. Nji, M. Ndi, I. Winkelmann, C. Von Ballmoos, D. Drew, Dissecting the proton transport pathway in electrogenic Na^+/H^+ antiporters. *Proc. Natl. Acad. Sci. U.S.A.* **114**, E1101–E1110 (2017).

37. E. Frumence, S. Genetet, P. Ripoche, A. Iolascon, I. Andolfo, C. L. Van Kim, Y. Colin, I. Mouro-Chanteloup, C. Lopez, Rapid CL^-/HCO_3^- exchange kinetics of AE1 in HEK293 cells and hereditary stomatocytosis red blood cells. *Am. J. Physiol. Cell Physiol.* **305**, 654–662 (2013).

38. J. D. Walter, M. Sawicka, R. Dutzler, Cryo-EM structures and functional characterization of murine Slc26a9 reveal mechanism of uncoupled chloride transport. *eLife* **8**, e46986 (2019).

39. A. T. Bozzi, L. B. Bane, W. A. Weihofen, A. Singharoy, E. R. Guillen, H. L. Ploegh, K. Schulten, R. Gaudet, Crystal structure and conformational change mechanism of a bacterial nramp-family divalent metal transporter. *Structure* **24**, 2102–2114 (2016).

40. C. Ma, Z. Hao, G. Huysmans, A. Lesiuk, P. Bullough, Y. Wang, M. Bartlam, S. E. Phillips, J. D. Young, A. Goldman, S. A. Baldwin, V. L. G. Postis, A versatile strategy for production of membrane proteins with diverse topologies: Application to investigation of bacterial homologues of human divalent metal ion and nucleoside transporters. *PLOS ONE* **10**, e0143010 (2015).

41. S. Antala, R. E. Dempski, The human ZIP4 transporter has two distinct binding affinities and mediates transport of multiple transition metals. *Biochemistry* **51**, 963–973 (2012).

42. K. M. Taylor, R. I. Nicholson, The LZT proteins; the LIV-1 subfamily of zinc transporters. *Biochim. Biophys. Acta* **1611**, 16–30 (2003).

43. E. M. Bafaro, M. W. Maciejewski, J. C. Hoch, R. E. Dempski, Concomitant disorder and high-affinity zinc binding in the human zinc- and iron-regulated transport protein 4 intracellular loop. *Protein Sci.* **28**, 868–880 (2019).

44. E. Park, R. Mackinnon, Structure of the CLC-1 chloride channel from *Homo Sapiens*. *eLife* **7**, e36629 (2018).

45. W. Kabsch, XDS. *Acta Crystallogr. D Biol. Crystallogr.* **66**, 125–132 (2010).

46. A. J. McCoy, R. W. Grosse-Kunstleve, P. D. Adams, M. D. Winn, L. C. Storoni, R. J. Read, Phaser crystallographic software. *J. Appl. Cryst.* **40**, 658–674 (2007).

47. P. Emsley, K. Cowtan, Coot: Model-building tools for molecular graphics. *Acta Crystallogr. D Biol. Crystallogr.* **60**, 2126–2132 (2004).

48. P. D. Adams, P. V. Afonine, G. Bunkóczi, V. B. Chen, I. W. Davis, N. Echols, J. J. Headd, L. W. Hung, G. J. Kapral, R. W. Grosse-Kunstleve, A. J. McCoy, N. W. Moriarty, R. Oeffner, R. J. Read, D. C. Richardson, J. S. Richardson, T. C. Terwilliger, P. H. Zwart, PHENIX: A comprehensive Python-

based system for macromolecular structure solution. *Acta Crystallogr. D Biol. Crystallogr.* **66**, 213–221 (2010).

49. T. I. Croll, ISOLDE: A physically realistic environment for model building into low-resolution electron-density maps. *Acta Crystallogr. D Struct. Biol.* **74**, 519–530 (2018).

50. A. T. Bozzi, C. M. Zimanyi, J. M. Nicoludis, B. K. Lee, C. H. Zhang, R. Gaudet, Structures in multiple conformations reveal distinct transition metal and proton pathways in an nramp transporter. *eLife* **8**, e41124 (2019).

51. C. A. Schneider, W. S. Rasband, K. W. Eliceiri, NIH Image to ImageJ: 25 years of image analysis. *Nat. Methods* **9**, 671–675 (2012).

52. S. Vilar, G. Cozza, S. Moro, Medicinal chemistry and the molecular operating environment (MOE): Application of QSAR and molecular docking to drug discovery. *Curr. Top. Med. Chem.* **8**, 1555–1572 (2008).

53. C. I. Bayly, K. M. Merz, D. M. Ferguson, W. D. Cornell, T. Fox, J. W. Caldwell, P. A. Kollman, P. Cieplak, I. R. Gould, D. C. Spellmeyer, A second generation force field for the simulation of proteins, nucleic acids, and organic molecules. *J. Am. Chem. Soc.* **117**, 5179–5197 (1995).

54. R Core Team, *R: A Language and Environment for Statistical Computing* (R Foundation for Statistical Computing, 2011), vol. 1.

55. H. Ashkenazy, S. Abadi, E. Martz, O. Chay, I. Mayrose, T. Pupko, N. Ben-Tal, ConSurf 2016: An improved methodology to estimate and visualize evolutionary conservation in macromolecules. *Nucleic Acids Res.* **44**, W344–W350 (2016).

56. J. Mistry, S. Chuguransky, L. Williams, M. Qureshi, G. A. Salazar, E. L. L. Sonnhammer, S. C. E. Tosatto, L. Paladin, S. Raj, L. J. Richardson, R. D. Finn, A. Bateman, Pfam: The protein families database in 2021. *Nucleic Acids Res.* **49**, D412–D419 (2021).

57. K. D. Tsirigos, C. Peters, N. Shu, L. Käll, A. Elofsson, The TOPCONS web server for consensus prediction of membrane protein topology and signal peptides. *Nucleic Acids Res.* **43**, W401–W407 (2015).

58. J. D. Thompson, D. G. Higgins, T. J. Gibson, CLUSTAL W: Improving the sensitivity of progressive multiple sequence alignment through sequence weighting, position-specific gap penalties and weight matrix choice. *Nucleic Acids Res.* **22**, 4673–4680 (1994).

59. I. Letunic, P. Bork, Interactive Tree of Life (iTOL) v4: Recent updates and new developments. *Nucleic Acids Res.* **47**, W256–W259 (2019).

60. E. Krissinel, K. Henrick, Inference of macromolecular assemblies from crystalline state. *J. Mol. Biol.* **372**, 774–797 (2007).

61. X. Yu, G. Yang, C. Yan, J. L. Baylon, J. Jiang, H. Fan, G. Lu, K. Hasegawa, H. Okumura, T. Wang, E. Tajkhorshid, S. Li, N. Yan, Dimeric structure of the uracil: Proton symporter UraA provides mechanistic insights into the SLC4/23/26 transporters. *Cell Res.* **27**, 1020–1033 (2017).

62. E. R. Geertsma, Y. N. Chang, F. R. Shaik, Y. Neldner, E. Pardon, J. Steyaert, R. Dutzler, Structure of a prokaryotic fumarate transporter reveals the architecture of the SLC26 family. *Nat. Struct. Mol. Biol.* **22**, 803–808 (2015).

63. Y. Alguel, S. Amillis, J. Leung, G. Lambrinidis, S. Capaldi, N. J. Scull, G. Craven, S. Iwata, A. Armstrong, E. Mikros, G. Diallinas, A. D. Cameron, B. Byrne, Structure of eukaryotic purine/H^+ symporter UapA suggests a role for homodimerization in transport activity. *Nat. Commun.* **7**, 11336 (2016).

64. H. M. Berman, J. Westbrook, Z. Feng, G. Gilliland, T. N. Bhat, H. Weissig, I. N. Shindyalov, P. E. Bourne, The Protein Data Bank. *Nucleic Acids Res.* **28**, 235–242 (2000).
